# A methodology for global validation of microarray experiments

**DOI:** 10.1186/1471-2105-7-333

**Published:** 2006-07-05

**Authors:** Mathieu Miron, Owen Z Woody, Alexandre Marcil, Carl Murie, Robert Sladek, Robert Nadon

**Affiliations:** 1 McGill University and Genome Quebec Innovation Centre, 740 avenue du Docteur Penfield, Montreal, Quebec, H3A 1A4, Canada; 2 McGill University Department of Human Genetics, 1205 avenue du Docteur Penfield N5/13, Montreal, Quebec, H3A 1B1, Canada

## Abstract

**Background:**

DNA microarrays are popular tools for measuring gene expression of biological samples. This ever increasing popularity is ensuring that a large number of microarray studies are conducted, many of which with data publicly available for mining by other investigators. Under most circumstances, validation of differential expression of genes is performed on a gene to gene basis. Thus, it is not possible to generalize validation results to the remaining majority of non-validated genes or to evaluate the *overall *quality of these studies.

**Results:**

We present an approach for the global validation of DNA microarray experiments that will allow researchers to evaluate the general quality of their experiment and to extrapolate validation results of a subset of genes to the remaining non-validated genes. We illustrate why the popular strategy of selecting only the most differentially expressed genes for validation generally fails as a global validation strategy and propose random-stratified sampling as a better gene selection method. We also illustrate shortcomings of often-used validation indices such as overlap of significant effects and the correlation coefficient and recommend the concordance correlation coefficient (CCC) as an alternative.

**Conclusion:**

We provide recommendations that will enhance validity checks of microarray experiments while minimizing the need to run a large number of labour-intensive individual validation assays.

## Background

Microarrays provide large-scale comparative gene expression profiles between biological samples by simultaneously detecting either expression or differential expression in thousands of genes. The lack of agreement among various technologies putatively measuring the same processes has prompted calls for microarray results to be validated with other technologies before they are published [1,2]. Microarray findings are usually validated on a gene-by-gene basis to lend support to biological models. It is not always clear, however, the extent to which the validation of these genes reflects the entire microarray experiment, in part because validation procedures often fall short of optimal sampling and statistical requirements [3]. Thus, microarray results where only a handful of genes have been validated, with little concern regarding the remainder of the data, are common. Moreover, public repositories of microarray experiments contain an ever increasing number of archived studies for which it is not readily possible to evaluate the quality. Global validation of microarray experiments is necessary to address these shortcomings. A global validation approach should provide an index of the quality of the fold-change estimates regarding all differentially expressed genes. Such an approach would be a valuable tool for investigators to assess the value of their microarray experiments. Furthermore, if routinely provided in archived gene expression experiments, global validation information would identify studies most useful for hypothesis generation and would also provide a study confidence index that could be used for several applications including meta-analysis [4] and Bayesian network modelling [5]. Three important aspects of global validation must be considered: what measurement should be validated, how should a subset of differentially expressed genes be chosen for follow-up, and what statistical evidence is needed to confirm validation of the microarray results in total?

It has been proposed that fold-change (FC), rather than raw expression, is the appropriate measure for comparing results across platforms [6,7]. Otherwise, various technology-specific artifacts (e.g., probe-specific biases across microarray platforms, amplification bias in PCR) can compromise direct comparisons between gene expression measurements. At the same time, there is general consensus that whereas FC is a reasonable measure of effect size, it is inadequate as a test-statistic [20].

Investigators may select genes for validation based on reagent availability or they may select genes based on *ad-hoc *or *a-priori *biological models. One common strategy is to select the largest FCs or the most statistically significant differentially expressed genes, based on the idea that large effects are more likely to be valid [8]. Such non-random sampling strategies have limited utility as gene selection procedures because validation results do not readily generalize to the entire set of differentially expressed genes. Moreover, the "regression toward the mean" statistical artifact [9,10], whose effects are exacerbated by selecting genes with the largest FCs, may lead to underestimating the global level of agreement between microarray and validation samples. We describe two random sampling strategies which provide data appropriate for global validation.

Finally, what statistical evidence is needed to assess global validation? Perfect agreement between two sets of FC measurements is indicated by correspondence of the paired data points to the identity line (regression slope of 1 with a y-intercept of 0). Extent of agreement between this identity line and the data is not captured, however, by the commonly-used Pearson *r *correlation coefficient. For example, high *r*^2 ^values could be obtained between two sets of observations even if the average of one set differed greatly from the other (good precision in predicting one set based on the other, but poor accuracy). By contrast, low *r*^2 ^values could be obtained, despite the two sets agreeing quite well on average (good accuracy in predicting one set based on the other, but poor precision). A better indicator of validation is provided by the concordance correlation coefficient (CCC) [11-13], which combines accuracy and precision coefficients in one index.

The CCC can vary from 1 (perfect agreement) to -1 (perfect reversed agreement), with zero representing no agreement. Its precision coefficient squared is the Pearson *r*^2^, which measures how close the data points are to the least-squares regression line; the CCC's accuracy coefficient measures how closely the regression line agrees with the identity line. Precision-squared and accuracy can vary from zero (no agreement) to 1 (perfect agreement). CCC values can be small because precision is low, accuracy is low, or both.

We present simulated and empirical (microarray and qrPCR) data to illustrate deficiencies in selecting only the largest effects for retest and to propose better gene selection methods. We also examine frequently-used statistical metrics for assessing validation and contrast their performance with the CCC index. We show that CCC is a useful predictor of global validation of microarray experiments, and that it can be used as an index of quality for all microarray studies.

## Results

### Performance of sampling strategies using simulated data

**Figure 1:**
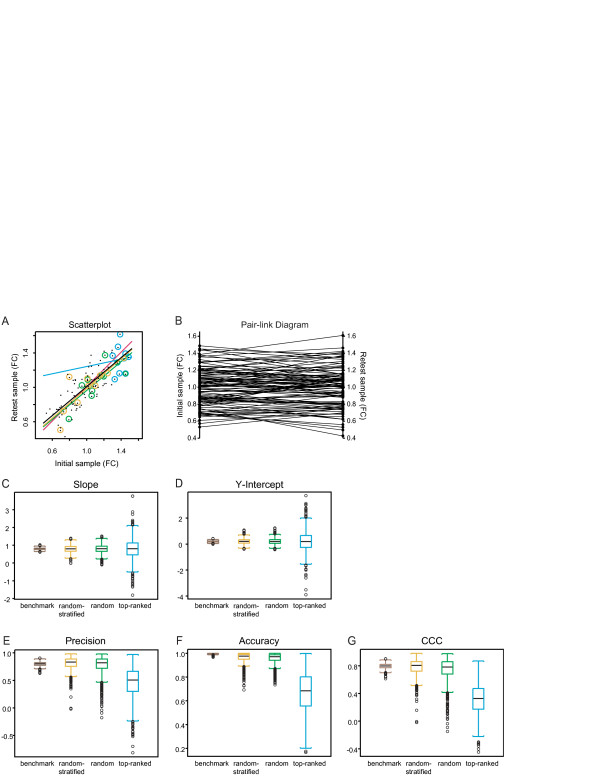
**Simulation of fold-change (FC) validation**. A scatter plot from one simulation iteration is shown in (A) where the selected genes are circled in orange (random-stratified), green (random) and blue (top-ranked). The regression lines are orange (random-stratified), green (random), blue (top-ranked), red (identity) and brown (full benchmark sample). (B) Pair-link plot which connects paired data points from (A); the tendency for the lines to cross illustrates regression toward the mean. Box plots of the benchmark and the various sampling methods for the 1000 simulations are shown for (C) slope, (D) y-intercept, (E) precision (Pearson *r*), (F) accuracy and (G) concordance correlation coefficient (CCC). The lines of the box plots are color matched as above.

The selection of a subset of genes for global validation is critical. To evaluate the effects of sampling, we compared three strategies (random-stratified, random, and top-ranked sampling) by generating 1000 simulated data sets each containing 100 upregulated genes. These 100 FCs were simulated to correlate approximately 0.80 with retest FCs; for each simulation run, 10 observations were selected for each sampling strategy (see Methods). The output from one of the simulation runs is presented in Figure 1A. For each simulation run, five measures were calculated for the full (benchmark) set of 100 observations and for each of the three sampling strategies: least-squares regression slopes and y-intercepts, CCCs (and the constituent accuracy and precision coefficients).

**Figure 2:**
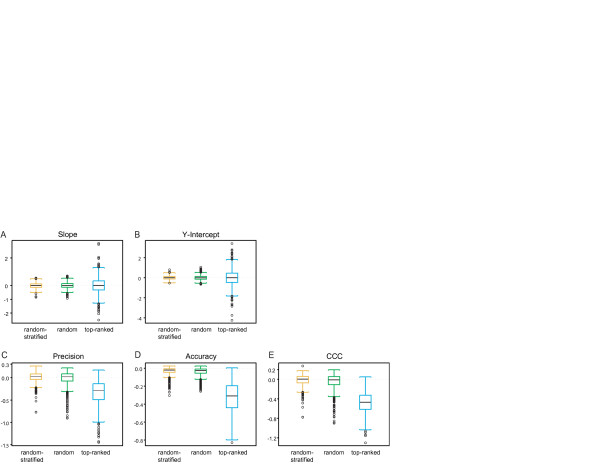
**Simulation of fold-change (FC) validation (difference boxplots)**. Box plots of differences (sampling method minus full benchmark sample) for the 1000 simulations are shown for (A) slope, (B) y-intercept, (C) precision (Pearson *r*), (D) accuracy and (E) concordance correlation coefficient (CCC). In each boxplot graph, the dotted line at 0 represents perfect agreement. The lines of the box plots represent: orange (random-stratified), green (random) and blue (top-ranked).

Boxplots of the statistical indices produced by the 100 benchmark values and by the 10 values for each of the three sampling strategies across the simulation runs are shown in Figures 1C–G (see also Additional file 1). Figures 2A–E display the 100 benchmark values subtracted from the calculated values for each sampling strategy, reflecting how closely the respective strategies approximate the ideal of validating all genes [see Additional file 1].

The top-ranked sampling procedure produced inferior estimates of all five statistical indices relative to random and random-stratified sampling. Slope and y-intercept values were accurately estimated across all sampling methods, although estimates for top-ranked sampling were highly variable (Figures 1C and 1D, 2A and 2B). Moreover, 6.8% of the slopes for the top-ranked samples were negative (i.e., in the opposite direction) compared to 0.02% (random-stratified) and 0.03% (random) (Figure 1C).

Top-ranked sampling also generated both highly inaccurate (downwardly biased) and highly variable estimates of precision, accuracy, and CCC compared to random-stratified and random sampling (Figures 1E,F, and 1G, 2C,D, and 2E, and Additional file 1). Moreover, only 32% of the top-ranked precision values were greater than 0.63 (the nominal *p *< 0.05, two-tailed significance level) compared to 93% and 87% for the random-stratified and random samples, respectively (uncorrected for multiple testing and assuming random sampling) (Figure 2C).

The extent of the bias in the precision estimates of both random and random-stratified sampling will vary with the true population correlation and with sample size. This bias should be, however, negligible in microarray validation studies. For random sampling, there are formulas which provide approximate corrections for the negative bias, which can be as high as 0.03 – 0.04 [14]. To estimate the size of the bias for the type of stratification in the present study, we conducted additional simulations (10,000 runs at a time) in which we varied the number of "genes" selected per stratum (1–9 of 10). For randomly stratified data, the upward bias (mean difference with the benchmark data) of the sample correlation coefficient ranged from a high of 0.0040 (*n *= 1 per stratum) to a low of 0.0003 (*n *= 9 per stratum).

In summary, random and random-stratified sampling performed similarly well, although random sampling was slightly more variable and produced more outliers on all indices (Figures 1C–G and Additional file 1). Moreover, the top-ranked sampling strategy performed substantially worse than either of the two other strategies.

### Performance of sampling strategies using empirical microarray data

**Figure 3:**
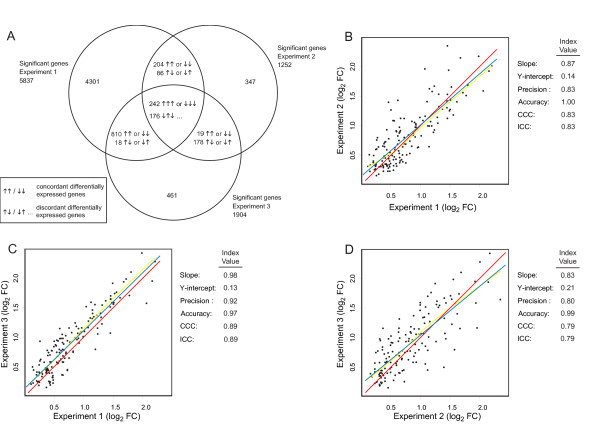
**Concordance between microarray experiments**. Overlap of statistically significant differentially expressed genes across the three repeated microarray experiments is shown in (A). Log_2 _fold-changes (FCs) for genes which were significantly upregulated in each of the three experiments were averaged across replicates within each experiment (*n *= 5 for Experiments 1 and 2; *n *= 4 for Experiment 3). Pairwise scatter plots among these averaged FCs are shown in (B-D). The regression lines are: the identity line (red), least-squares (blue) and loess (yellow).

We performed three identical replicate experiments with mouse NIH 3T3-L1 preadipocytes treated or not with the steroid hormone dexamethasone (DEX) for 3 h prior to harvesting. Labelled RNA from each experiment was hybridized to Affymetrix MG U74Av2 microarrays (see Methods and the MIAME document online). The overlap of statistically significantly differentially expressed genes among the three experiments is displayed in a Venn diagram (Figure 3A). A substantial number of genes were statistically significant in opposite directions across the experiments, despite setting the false discovery rate (FDR) at 0.05 within each experiment. Moreover, many more genes were significant in Experiment 1 because variability among replicates was substantially lower than in the other two experiments [see Additional file 2]. One-hundred and fifty genes were significantly upregulated in all three experiments (see Methods). Good agreement was observed among the three microarray replicate experiments for these genes (Figures 3B–D).

To further examine differences in random-stratified versus top-ranked sampling, we selected 29 of the 150 upregulated genes to validate with qrPCR: the top 15 differentially expressed genes and 14 genes from a random stratification scheme (Table 1; also see Methods and Additional file 8). The 15 top-ranked genes and nine of the 14 genes selected by random stratification were significantly differentially expressed by qrPCR in all three experiments. Of the remaining five genes in the stratified sample, three were significant in two experiments (*p *< 0.005) and two were significant in one experiment (*p *< 0.05; see Table 1 and Additional files 1 and 8).

**Figure 4:**
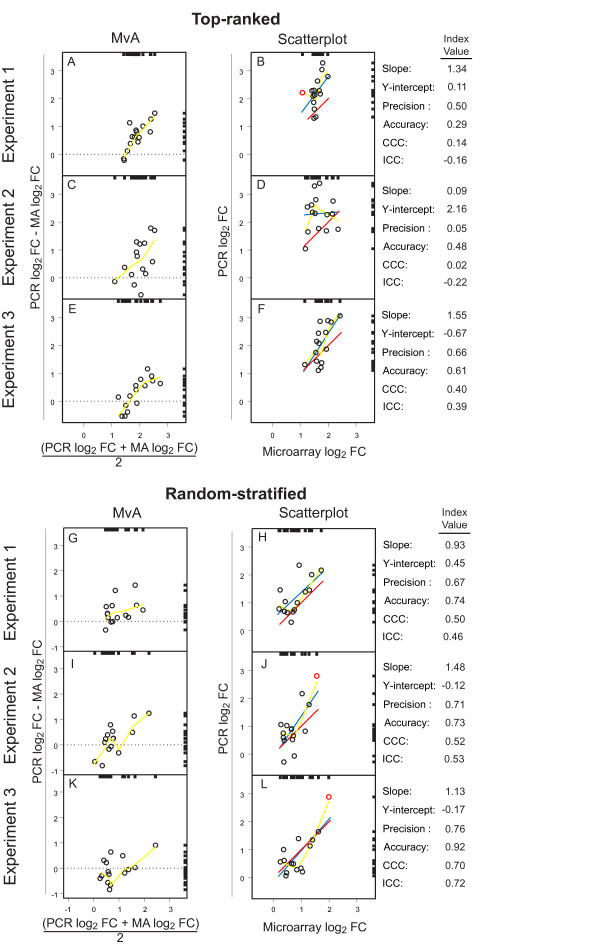
**Validation of microarray experiments by qrPCR**. Concordance between microarray and qrPCR FCs (averaged across within-experiment replicates) for top-ranked (*n *= 15) and random-stratified sampling methods (*n *= 15) for each microarray experiment is shown in the six scatter plots on the right (B, D, F, H, J, L); corresponding MvA plots are shown on the left (A, C, E, G, I, K). The log_2 _fold-change (FC) values for qrPCR were determined from the calibration curve performed for each selected gene. Deleting the red data points in panels B, J, and L, which had large influence on the regression slope (standardized slope *dfBeta *values > 1) had little effect on the CCC values (data not shown). Results of a robust regression approach for detecting curvature in H, J, and L yielded inconsistent results; *p *> 0.10 (H and J) and *p *< 0.10 (L) (see Methods); least squares regression tests for quadratic effects were likewise inconsistent; *p *values for the quadratic term for H, J, and L were 0.28, 0.08, and 0.004, respectively. The regression lines are: the identity line (red), least-squares (blue) and loess (yellow). Random-stratified sampling consistently yields better results than top-ranked sampling.

Figure 4 presents validation results from the three microarray experiments with aliquots of the same RNA samples (the type of technical validation that is typically reported). As with the simulation findings, scatter plots of each experiment show that gene selection by stratification yields more accurate and less variable precision and accuracy coefficients compared to top-ranked sampling, with correspondingly higher CCC estimates. As a point of comparison to the CCC index, we also report intraclass correlation [15,16] values.

**Figure 5:**
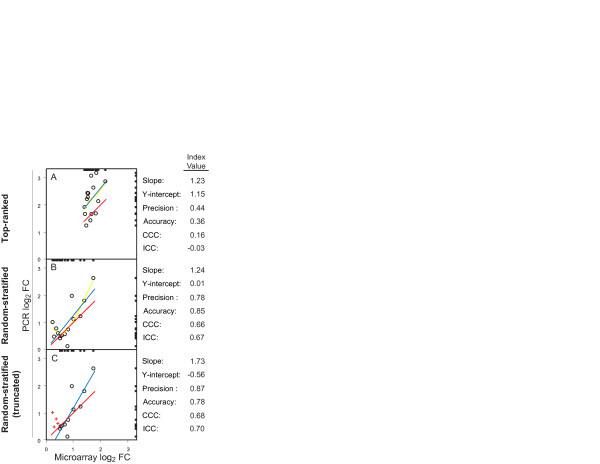
**Validation of microarray experiments by qrPCR (data averaged across experiments)**. Data in (A) were averaged across Figures 4B, D, and F; data in (B) were averaged across Figures 4H, J, and L. (C) Same as in (B) with log_2 _FCs < 0.5 (red +'s) removed. The regression lines are: the identity line (red), least-squares (blue) and loess (yellow).

To obtain more stable estimates of the various indices, we averaged the data across the three experiments (Figures 5A–B). Compared to sampling top-ranked genes, random-stratified sampling was 3.14 times more precise (0.78^2 ^vs. 0.44^2^) and 2.36 times more accurate (0.85 vs. 0.36). Moreover, the CCC was robust to departures from linearity (Figure 5C), influential regression data points (Figures 4B,J, and 4L) and log2 FCs < 0.5 [see Additional file 6].

**Figure 6:**
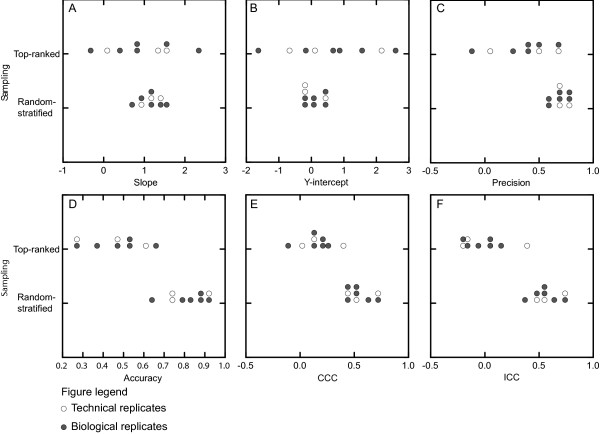
**Dot plots of the values for the various validation indices for technical and biological validation samples**. Dot plots of (A) slope, (B) y-intercept, (C) precision (Pearson *r*), (D) accuracy (E) concordance correlation coefficient (CCC) and (F) intraclass correlation coefficient (ICC). In each dot plot, the dots represent the comparisons between technical (open dots) and biological replicates (filled dots) of microarray to qrPCR experiments. As with the simulated data, the slope and y-intercept results for the top-ranked comparisons are unbiased relative to random-stratified comparisons, but they are more variable. The precision, accuracy, CCC and ICC estimates are, similar to the simulated data, more variable and downwardly biased.

Results from each microarray experiment were also compared to the qrPCR results obtained from the RNA samples from the two other experiments (biological validation), yielding similar results [Additional files 3 and 4]. Figure 6 shows the distributions of the various indices for the technical and biological validations. Contrary to expectation, there are no obvious differences between the technical and biological validation results.

### Relation between microarray and qrPCR

**Figure 7:**
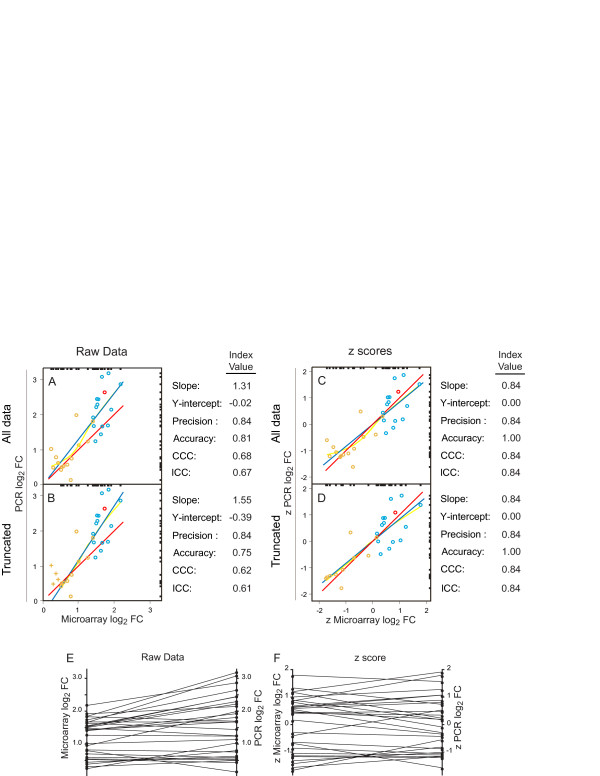
**Validation of microarray experiments by qrPCR (data averaged across experiments; top-ranked and random-stratified data combined)**. Data in (A) were combined from Figures 5A and B; (B) Same as in (A) with log_2 _FCs < 0.5 (yellow +'s) removed. (C-D) Standardized (z-scores) data from A-B. (E) Pair-link diagram of data from panel A; apparent regression away from the mean is evident by the mostly positive line slopes, especially among the larger microarray FCs. (F) Pair-link diagram of data from panel C; regression toward the mean is evident by the tendency of the lines to cross. Blue data points in all graphs are from the top-ranked sampling method; orange points are from the random-stratified method; the single red point was included in both samples. The regression lines are: the identity line (red), least-squares (blue) and loess (yellow).

Predicted qrPCR FCs were larger than microarray FCs, an effect which increased with increasing FCs, as indicated by the least-squares regression (blue) lines being above the identity (red) lines for most of the data range in Figures 4, 5. This pattern is also evident in Figures 7A–B, which combine the data from Figures 5A–B. We note also that we obtained better validation results (especially with the precision index) with qrPCR for both sampling strategies when qrPCR data were calibrated by the standard curve method rather than assuming 2-fold amplification of the PCR reaction using the CT measurements (compare Figures 4 and 5 with Additional files 5 and 7, respectively). Also, the robust multichip analysis (RMA, [17]) used in our analysis is one of many algorithms available for normalizing Affymetrix data. Comparison of results with other normalization algorithms is beyond the scope of the study and is complicated by the fact that genes were selected for subsequent validation based on the RMA data. Caveats notwithstanding, we provide validation indices for other popular normalization algorithms [dChip (with and without mismatches) [18], GC-RMA [19], MAS 5.0 [20], and PLIER [21]. The MAS 5.0 FC estimates produced the highest concordance with the qrPCR FC estimates, with CCC, precision and accuracy values of 0.77, 0.85 and 0.91 for random-stratified sampling and 0.69, 0.73 and 0.95 for top-ranked sampling, respectively [Additional file 1].

### Regression toward the mean

The lower precision and accuracy of the top sampling strategy can be explained by the regression toward the mean phenomenon. The phenomenon describes the tendency for extreme values of one set of observations to be less extreme on a second set. The lower the true correlation and the more extreme the values on the initial set, the more pronounced the tendency. Note also that regression toward the mean is bidirectional; the artifact remains if the initial and the retest sets are reversed.

The regression toward the mean effect depends solely on the correlation between two sets of observations; it occurs whenever the correlation is less than perfect (i.e., *r*^2 ^< 1) [9,10]. This correlation in turn depends on the variability of the true (unknown) values and the variability of the random error associated with the measurements. Reducing random error (e.g., by stringent quality control procedures) and sampling across the entire data range maximizes the observed correlation and minimizes the adverse effects of regression toward the mean. Finally, although for ease of exposition we conducted our simulations assuming linearity and normally distributed random error, the regression toward the mean phenomenon does not depend on these assumptions [9,10].

The strength of the adverse effect of top-ranked sampling will depend on the distributions of the microarray and validated sample data. Accordingly, choice of microarray and qrPCR preprocessing methods (e.g., background correction, normalization, transformation, calibration) will affect the regression toward the mean effect to the extent that they affect the data distributions, although the effect will always be present to some degree. Assuming linearity and homoscedasticity, restricting the microarray data to the top-ranked effects will underestimate the population correlation between microarray (*x*) and validation samples (*y*) according to the following formula [22]:

ρxTop−ranked,y=(σxTop−rankedσxFull−range)ρxFull−range,y(σxTop−rankedσxFull−range)ρxFull−range,y2+1
 MathType@MTEF@5@5@+=feaafiart1ev1aaatCvAUfKttLearuWrP9MDH5MBPbIqV92AaeXatLxBI9gBaebbnrfifHhDYfgasaacH8akY=wiFfYdH8Gipec8Eeeu0xXdbba9frFj0=OqFfea0dXdd9vqai=hGuQ8kuc9pgc9s8qqaq=dirpe0xb9q8qiLsFr0=vr0=vr0dc8meaabaqaciaacaGaaeqabaqabeGadaaakeaaiiGacqWFbpGCdaWgaaWcbaGaemiEaG3aaSbaaWqaaiabdsfaujabd+gaVjabdchaWjabgkHiTiabdkhaYjabdggaHjabd6gaUjabdUgaRjabdwgaLjabdsgaKbqabaWccqGGSaalcqWG5bqEaeqaaOGaeyypa0ZaaSaaaeaadaqadiqaamaaliaabaGae83Wdm3aaSbaaSqaaiabdIha4naaBaaameaacqWGubavcqWGVbWBcqWGWbaCcqGHsislcqWGYbGCcqWGHbqycqWGUbGBcqWGRbWAcqWGLbqzcqWGKbazaeqaaaWcbeaaaOqaaiab=n8aZnaaBaaaleaacqWG4baEdaWgaaadbaGaemOrayKaemyDauNaemiBaWMaemiBaWMaeyOeI0IaemOCaiNaemyyaeMaemOBa4Maem4zaCMaemyzaugabeaaaSqabaaaaaGccaGLOaGaayzkaaGae8xWdi3aaSbaaSqaaiabdIha4naaBaaameaacqWGgbGrcqWG1bqDcqWGSbaBcqWGSbaBcqGHsislcqWGYbGCcqWGHbqycqWGUbGBcqWGNbWzcqWGLbqzaeqaaSGaeiilaWIaemyEaKhabeaaaOqaamaakaaabaWaaeWaceaadaWccaqaaiab=n8aZnaaBaaaleaacqWG4baEdaWgaaadbaGaemivaqLaem4Ba8MaemiCaaNaeyOeI0IaemOCaiNaemyyaeMaemOBa4Maem4AaSMaemyzauMaemizaqgabeaaaSqabaaakeaacqWFdpWCdaWgaaWcbaGaemiEaG3aaSbaaWqaaiabdAeagjabdwha1jabdYgaSjabdYgaSjabgkHiTiabdkhaYjabdggaHjabd6gaUjabdEgaNjabdwgaLbqabaaaleqaaaaaaOGaayjkaiaawMcaaiab=f8aYnaaDaaaleaacqWG4baEdaWgaaadbaGaemOrayKaemyDauNaemiBaWMaemiBaWMaeyOeI0IaemOCaiNaemyyaeMaemOBa4Maem4zaCMaemyzaugabeaaliabcYcaSiabdMha5bqaaiabikdaYaaakiabgUcaRiabigdaXaWcbeaaaaaaaa@AF95@

Where ρ and σ are the population correlations and standard deviations, respectively.

Regression toward the mean can be most easily illustrated when the two sample means and standard deviations are at least approximately equal, as is the case for the simulated data in Figure 1 and the microarray-microarray data in Figure 3. Note the larger distances between the identity (red) line in Figure 1A and the benchmark data least squares regression (black) line for the extreme (low and high) values of the initial sample (x-axis). Similar differences are seen between the identity (red) line and the least squares regression (blue) line in Figures 3B–D. For low initial sample values, predicted values on retest are larger; for large initial sample values, predicted values on retest are smaller.

Pair-link diagrams provide another graphical illustration [9] (Figure 1B). The lines linking the initial scores to their respective retest scores tend to cross. On average, high scores on the initial sample have negative (decreasing) slopes, low scores have positive (increasing) slopes, and middle scores have flat slopes. The upshot of this tendency is that retest values will have lower precision and lower accuracy when top-ranked initial values are selected for retest.

By contrast, the pair-link diagram for the microarray/qrPCR data (Figure 7E) shows that most lines linking the microarray FCs to their respective qrPCR FCs have a positive slope, especially among the top-ranked microarray values. Despite appearances, regression toward the mean is nonetheless present and provides an explanation for the lower level of agreement observed among the top-ranked genes. This type of apparent "regression away from the mean" can only occur when the standard deviation of the validation sample is larger than the standard deviation of the initial sample, as here (sd_qrPCR _= 0.85; sd_Microarray _= 0.56). Regression toward the mean, however, is a phenomenon of standardized scores (it is simply not necessary for illustration purposes to use standardized scores when standard deviations are equal). When variance is taken into account and measurements are converted to standardized z-scores, the regression toward the mean effect is evident (Figures 7C, D and F). Extreme standardized qrPCR FCs are less extreme than their corresponding microarray FCs. Larger sample sizes would be needed to determine if the lower agreement among the top-ranked genes is due solely to regression toward the mean or to regression toward the mean plus some other effect (e.g., non-linearity).

## Discussion

Routine global validation of microarray results would provide valuable information on the quality of microarray studies and would complement existing standards for validating individual genes. Our results demonstrate that the outcome of global validation depends on how a subset of genes is chosen. Random-stratified sampling provides more accurate and more precise estimates of agreement between microarrays and qrPCR than does the often-used top-ranked sampling procedure. Our empirical results confirm the theoretical argument that selecting top-ranked differentially expressed genes for validation leads to underestimating the level of agreement between microarray and qrPCR validation FC values.

Genes which are deemed especially important to the experimenter can be validated separately from genes required for validation of the microarray experiment in total. Appreciation for the regression toward the mean effect, however, is still necessary for informed decisions regarding these specific genes. The tendency for extreme FCs to be less extreme on validation will still be operating, although the non-random sampling will make it difficult to assess the extent of the effect.

### CCC index of validation

We argue that a one-to-one correspondence between microarray and validation FC estimates is the gold standard for validation. This tight clustering of retest FC values around the identity line is indexed uniquely among validation indices by the CCC measure. Accordingly, the CCC provides dimensionless metrics with which to compare technology platforms, statistical procedures, and laboratory protocols, and ultimately, the overall quality of any given microarray study. In the case of unusually low CCC values, the accuracy and precision components provide clues on how the validation samples deviate from the standard which may in turn suggest procedural or statistical remedies. Regression slope and intercept estimates provide additional information to convert microarray FC estimates into estimates from lower throughput methods. Finally, the reported robustness of the CCC with as few as 10 data points [11] is supported in our data, as influential (outlier) data points and deviations from linearity had little effect on the CCC estimates, although robust analogues of the CCC are also available [23].

### Sampling

The relatively high CCC values we observed in pairwise comparisons between our microarray experiments (Figures 3B–D) lend support to FC as a good index of effect size for platform comparison purposes. However, FC measurements present a statistical technical difficulty when attempting to validate the entire FC range, including non-differentially expressed genes. Most log FCs near zero represent non-differentially expressed genes whose variation merely reflects noise. Correlation with qrPCR for these genes will be close to zero and the least squares line will be flat within this range. Differentially expressed genes, on the other hand, will have positive slopes for both up and down regulated genes. If only differentially expressed genes are selected for validation, up and down-regulated genes should be examined separately. Analyzing them together will upwardly bias correlation values due to a "range enhancement" artifact [22]; in extreme cases, the correlation between microarray and qrPCR FCs may be close to 1, despite zero correlations when up and down-regulated genes are analyzed separately.

The adverse effects of regression toward the mean are sometimes avoided when specific genes of interest are selected and they cover the full FC range coincidentally. The strategy remains less than optimal as a global validation strategy, however, because the non-random sampling nonetheless prevents the generalization of the conclusions to the remaining majority of differentially expressed genes.

### Threshold index of validation

All top-ranked, but only nine out of the 14 random-stratified genes in our study were statistically significant by qrPCR in the three samples. Non-significant *p *values tended to occur among the smaller FCs, but this effect was not uniform, as the smallest average microarray FC gene was significant in all three PCR samples. The seemingly paradoxical difference between the statistical significance threshold and the CCC approaches to validation can be explained as follows.

One difficulty with this type of threshold-based strategy is the choice of threshold. It can be shown that the smaller the initial *p *value, the more probable a second test will meet a specified probability threshold [24,25]. A true positive gene that is differentially expressed at *p *= 0.05 has only a 50% chance of being differentially expressed at *p *< 0.05 on retest; at *p *= 0.005, the probability of obtaining *p *< 0.05 on retest increases to 80% [24]. Accordingly, the larger its initial FC, the more likely the gene will exceed the decision threshold in the validation sample despite regression toward the mean. This threshold approach to validation, however, is adequate only if one is interested in the largest FC effects to the exclusion of more moderate but potentially important effects.

Additionally, consider the following example. Using a *p *< 0.05 threshold, a gene that is differentially expressed at *p *= 0.049 by microarray but at *p *= 0.051 by qrPCR would be said to not have validated despite almost identical *p *values. By contrast, a gene that is differentially expressed at *p *= 0.0001 by microarray and at *p *= 0.049 by qrPCR would be said to have validated despite a large discrepancy in *p *values. One way around this difficulty would be to require that the effect size of the validated sample not be significantly different from the effect size of the initial sample for a conclusion that the gene was validated, although this raises the additional question of how many replicate samples would be needed to provide sufficient statistical power for detecting differences [26]. Moreover, the issue arises whether a gene would be considered validated if it were significant in both initial and validated samples but with significantly different effect sizes in the two samples. Despite their limitations however, threshold approaches do provide a framework for dichotomous decisions regarding whether or not an individual gene has been validated.

The question arises of how one can reach this type of decision on individual genes with the validation approach that we are advocating. One approach might be to first determine the extent of validation across a number of appropriately (e.g., random, random stratified) sampled genes by some index of global validation (e.g., CCC). If the results of global validation were found to be inadequate, then the microarray experiment might well be considered a failure and the validation of individual genes moot. If on the other hand global validation were found to be adequate, then regression diagnostic methods might be used to identify outlier genes whose validation results deviated from the pattern of the majority of genes for a variety of reasons (e.g., splice variants; cross-hybridization).

These regression outlier genes could then be investigated further to determine their status; non-outlier genes would be considered validated.

A corollary to this approach is that one could extend the conclusion of validity to those microarray findings not selected for validation with PCR but whose effects fall within the sampled (and validated) range. Our approach advocates selection of genes through a random process; therefore, any index of global validation should be uninfluenced by the specific sample selected and should generalize to the non-validated genes. Moreover, the relative proportions of outlier and non-outlier genes would provide an indication of the relative risk involved in making such a generalization.

## Conclusion

Our results point to the importance of gene selection strategy, choice of qrPCR calibration methods, and choice of validation index in the assessment of microarray validation results. Sample sizes of 10 to 25 genes should be adequate for most validation purposes, although more observations may be needed to reliably estimate non-linearity between microarray and validation FC values. The CCC provides a global indication of the reproducibility of gene expression FCs estimated by microarrays, providing that a suitably random procedure is used to select genes for validation. Thus, we propose that the CCC be used as a universal measure of study quality.

## Methods

### Simulated data

FC values were simulated according to the following model: *FC *= *μ *+ *ε*, where *μ *~ *N *(1, 0.2) and *ε *~ *N *(0, 0.1). That is, for each of the 100 simulated genes, a "true" FC was randomly generated from a *N *(1, 0.2) distribution. Random error for each of these true values was randomly generated twice from a *N *(0, 0.1) distribution. Each of the pair of random error values was added to a single true value to produce an "initial sample" and a "retest sample" value. The 0.2 standard deviation across the true FC values and the 0.1 standard deviation value for *ε *were selected to produce an expected correlation of 0.80. The expected correlation between two measurements with iid error associated with the same true value (here FC) is the ratio of the variance of the true scores to the variance of the measured scores as follows (see [16] pp. 134–140):

E(r)=σT2σM2=σT2σT2+σε2
 MathType@MTEF@5@5@+=feaafiart1ev1aaatCvAUfKttLearuWrP9MDH5MBPbIqV92AaeXatLxBI9gBaebbnrfifHhDYfgasaacH8akY=wiFfYdH8Gipec8Eeeu0xXdbba9frFj0=OqFfea0dXdd9vqai=hGuQ8kuc9pgc9s8qqaq=dirpe0xb9q8qiLsFr0=vr0=vr0dc8meaabaqaciaacaGaaeqabaqabeGadaaakeaacqWGfbqrdaqadiqaaiabdkhaYbGaayjkaiaawMcaaiabg2da9maalaaabaacciGae83Wdm3aa0baaSqaaiabdsfaubqaaiabikdaYaaaaOqaaiab=n8aZnaaDaaaleaacqWGnbqtaeaacqaIYaGmaaaaaOGaeyypa0ZaaSaaaeaacqWFdpWCdaqhaaWcbaGaemivaqfabaGaeGOmaidaaaGcbaGae83Wdm3aa0baaSqaaiabdsfaubqaaiabikdaYaaakiabgUcaRiab=n8aZnaaDaaaleaacqWF1oqzaeaacqaIYaGmaaaaaaaa@48A2@

where σT2
 MathType@MTEF@5@5@+=feaafiart1ev1aaatCvAUfKttLearuWrP9MDH5MBPbIqV92AaeXatLxBI9gBaebbnrfifHhDYfgasaacH8akY=wiFfYdH8Gipec8Eeeu0xXdbba9frFj0=OqFfea0dXdd9vqai=hGuQ8kuc9pgc9s8qqaq=dirpe0xb9q8qiLsFr0=vr0=vr0dc8meaabaqaciaacaGaaeqabaqabeGadaaakeaaiiGacqWFdpWCdaqhaaWcbaGaemivaqfabaGaeGOmaidaaaaa@30C6@, σM2
 MathType@MTEF@5@5@+=feaafiart1ev1aaatCvAUfKttLearuWrP9MDH5MBPbIqV92AaeXatLxBI9gBaebbnrfifHhDYfgasaacH8akY=wiFfYdH8Gipec8Eeeu0xXdbba9frFj0=OqFfea0dXdd9vqai=hGuQ8kuc9pgc9s8qqaq=dirpe0xb9q8qiLsFr0=vr0=vr0dc8meaabaqaciaacaGaaeqabaqabeGadaaakeaaiiGacqWFdpWCdaqhaaWcbaGaemyta0eabaGaeGOmaidaaaaa@30B8@, and σε2
 MathType@MTEF@5@5@+=feaafiart1ev1aaatCvAUfKttLearuWrP9MDH5MBPbIqV92AaeXatLxBI9gBaebbnrfifHhDYfgasaacH8akY=wiFfYdH8Gipec8Eeeu0xXdbba9frFj0=OqFfea0dXdd9vqai=hGuQ8kuc9pgc9s8qqaq=dirpe0xb9q8qiLsFr0=vr0=vr0dc8meaabaqaciaacaGaaeqabaqabeGadaaakeaaiiGacqWFdpWCdaqhaaWcbaGae8xTdugabaGaeGOmaidaaaaa@3137@ are the variances of the true scores, the measured scores, and random error, respectively.

For the current simulated data,

E(r)=0.220.22+0.12=0.80.
 MathType@MTEF@5@5@+=feaafiart1ev1aaatCvAUfKttLearuWrP9MDH5MBPbIqV92AaeXatLxBI9gBaebbnrfifHhDYfgasaacH8akY=wiFfYdH8Gipec8Eeeu0xXdbba9frFj0=OqFfea0dXdd9vqai=hGuQ8kuc9pgc9s8qqaq=dirpe0xb9q8qiLsFr0=vr0=vr0dc8meaabaqaciaacaGaaeqabaqabeGadaaakeaacqWGfbqrdaqadiqaaiabdkhaYbGaayjkaiaawMcaaiabg2da9maalaaabaGaeGimaaJaeiOla4IaeGOmaiZaaWbaaSqabeaacqaIYaGmaaaakeaacqaIWaamcqGGUaGlcqaIYaGmdaahaaWcbeqaaiabikdaYaaakiabgUcaRiabicdaWiabc6caUiabigdaXmaaCaaaleqabaGaeGOmaidaaaaakiabg2da9iabicdaWiabc6caUiabiIda4iabicdaWiabc6caUaaa@441C@

### Sampling methods

Sampling from simulated or from microarray data was performed in three different manners. For top-ranked sampling, 10 (simulated data) or 15 (microarray data) largest up-regulated FCs were selected. For random sampling, 10 (or 15) observations were randomly selected from the entire range of upregulated genes. For random-stratified sampling, the entire list of upregulated genes was rank ordered according to FCs; the data were then divided into 10 (or 15) equal-sized bins and one gene per bin was selected randomly.

### Validation indices

The slope and y-intercept indices were estimated by least squares linear regression [27]. The formula for the CCC [11-13] is

rc=2rsysxsy2+sx2+(y¯−x¯)2.
 MathType@MTEF@5@5@+=feaafiart1ev1aaatCvAUfKttLearuWrP9MDH5MBPbIqV92AaeXatLxBI9gBaebbnrfifHhDYfgasaacH8akY=wiFfYdH8Gipec8Eeeu0xXdbba9frFj0=OqFfea0dXdd9vqai=hGuQ8kuc9pgc9s8qqaq=dirpe0xb9q8qiLsFr0=vr0=vr0dc8meaabaqaciaacaGaaeqabaqabeGadaaakeaacqWGYbGCdaWgaaWcbaGaem4yamgabeaakiabg2da9maalaaabaGaeGOmaiJaemOCaiNaem4Cam3aaSbaaSqaaiabdMha5bqabaGccqWGZbWCdaWgaaWcbaGaemiEaGhabeaaaOqaaiabdohaZnaaDaaaleaacqWG5bqEaeaacqaIYaGmaaGccqGHRaWkcqWGZbWCdaqhaaWcbaGaemiEaGhabaGaeGOmaidaaOGaey4kaSYaaeWaceaacuWG5bqEgaqeaiabgkHiTiqbdIha4zaaraaacaGLOaGaayzkaaWaaWbaaSqabeaacqaIYaGmaaGccqGGUaGlaaaaaa@4AE2@

where *r *is the Pearson correlation coefficient, sy2
 MathType@MTEF@5@5@+=feaafiart1ev1aaatCvAUfKttLearuWrP9MDH5MBPbIqV92AaeXatLxBI9gBaebbnrfifHhDYfgasaacH8akY=wiFfYdH8Gipec8Eeeu0xXdbba9frFj0=OqFfea0dXdd9vqai=hGuQ8kuc9pgc9s8qqaq=dirpe0xb9q8qiLsFr0=vr0=vr0dc8meaabaqaciaacaGaaeqabaqabeGadaaakeaacqWGZbWCdaqhaaWcbaGaemyEaKhabaGaeGOmaidaaaaa@30B5@ and y¯
 MathType@MTEF@5@5@+=feaafiart1ev1aaatCvAUfKttLearuWrP9MDH5MBPbIqV92AaeXatLxBI9gBaebbnrfifHhDYfgasaacH8akY=wiFfYdH8Gipec8Eeeu0xXdbba9frFj0=OqFfea0dXdd9vqai=hGuQ8kuc9pgc9s8qqaq=dirpe0xb9q8qiLsFr0=vr0=vr0dc8meaabaqaciaacaGaaeqabaqabeGadaaakeaacuWG5bqEgaqeaaaa@2E3F@ and sx2
 MathType@MTEF@5@5@+=feaafiart1ev1aaatCvAUfKttLearuWrP9MDH5MBPbIqV92AaeXatLxBI9gBaebbnrfifHhDYfgasaacH8akY=wiFfYdH8Gipec8Eeeu0xXdbba9frFj0=OqFfea0dXdd9vqai=hGuQ8kuc9pgc9s8qqaq=dirpe0xb9q8qiLsFr0=vr0=vr0dc8meaabaqaciaacaGaaeqabaqabeGadaaakeaacqWGZbWCdaqhaaWcbaGaemiEaGhabaGaeGOmaidaaaaa@30B3@ and x¯
 MathType@MTEF@5@5@+=feaafiart1ev1aaatCvAUfKttLearuWrP9MDH5MBPbIqV92AaeXatLxBI9gBaebbnrfifHhDYfgasaacH8akY=wiFfYdH8Gipec8Eeeu0xXdbba9frFj0=OqFfea0dXdd9vqai=hGuQ8kuc9pgc9s8qqaq=dirpe0xb9q8qiLsFr0=vr0=vr0dc8meaabaqaciaacaGaaeqabaqabeGadaaakeaacuWG4baEgaqeaaaa@2E3D@ are the *y *and *x *sample variances and means, respectively.

Note that *r*_*c *_= *r *when y¯
 MathType@MTEF@5@5@+=feaafiart1ev1aaatCvAUfKttLearuWrP9MDH5MBPbIqV92AaeXatLxBI9gBaebbnrfifHhDYfgasaacH8akY=wiFfYdH8Gipec8Eeeu0xXdbba9frFj0=OqFfea0dXdd9vqai=hGuQ8kuc9pgc9s8qqaq=dirpe0xb9q8qiLsFr0=vr0=vr0dc8meaabaqaciaacaGaaeqabaqabeGadaaakeaacuWG5bqEgaqeaaaa@2E3F@ = x¯
 MathType@MTEF@5@5@+=feaafiart1ev1aaatCvAUfKttLearuWrP9MDH5MBPbIqV92AaeXatLxBI9gBaebbnrfifHhDYfgasaacH8akY=wiFfYdH8Gipec8Eeeu0xXdbba9frFj0=OqFfea0dXdd9vqai=hGuQ8kuc9pgc9s8qqaq=dirpe0xb9q8qiLsFr0=vr0=vr0dc8meaabaqaciaacaGaaeqabaqabeGadaaakeaacuWG4baEgaqeaaaa@2E3D@ and *s*_*y *_= *s*_*x *_.

The CCC can also be written as the product of the accuracy and the precision coefficients. The precision coefficient is given by:

r=∑zxzyN
 MathType@MTEF@5@5@+=feaafiart1ev1aaatCvAUfKttLearuWrP9MDH5MBPbIqV92AaeXatLxBI9gBaebbnrfifHhDYfgasaacH8akY=wiFfYdH8Gipec8Eeeu0xXdbba9frFj0=OqFfea0dXdd9vqai=hGuQ8kuc9pgc9s8qqaq=dirpe0xb9q8qiLsFr0=vr0=vr0dc8meaabaqaciaacaGaaeqabaqabeGadaaakeaacqWGYbGCcqGH9aqpdaWcaaqaamaaqaeabaGaemOEaO3aaSbaaSqaaiabdIha4bqabaGccqWG6bGEdaWgaaWcbaGaemyEaKhabeaaaeqabeqdcqGHris5aaGcbaGaemOta4eaaaaa@38B7@

Where *z *are standardized scores (mean of zero with unit variance).

The accuracy coefficient is given by:

χa=2ϖ+1ϖ+ν2, whereν2=(y¯−x¯)2sysxand ϖ=sysx.
 MathType@MTEF@5@5@+=feaafiart1ev1aaatCvAUfKttLearuWrP9MDH5MBPbIqV92AaeXatLxBI9gBaebbnrfifHhDYfgasaacH8akY=wiFfYdH8Gipec8Eeeu0xXdbba9frFj0=OqFfea0dXdd9vqai=hGuQ8kuc9pgc9s8qqaq=dirpe0xb9q8qiLsFr0=vr0=vr0dc8meaabaqaciaacaGaaeqabaqabeGadaaakqaabeqaaGGaciab=D8aJnaaBaaaleaacqWGHbqyaeqaaOGaeyypa0ZaaSaaaeaacqaIYaGmaeaacqWFwpGDcqGHRaWkdaWcaaqaaiabigdaXaqaaiab=z9a2baacqGHRaWkcqWF9oGBdaahaaWcbeqaaiabikdaYaaaaaGccqGGSaalcqqGGaaicqqG3bWDcqqGObaAcqqGLbqzcqqGYbGCcqqGLbqzaeaacqWF9oGBdaahaaWcbeqaaiabikdaYaaakiabg2da9maalaaabaWaaeWaceaacuWG5bqEgaqeaiabgkHiTiqbdIha4zaaraaacaGLOaGaayzkaaWaaWbaaSqabeaacqaIYaGmaaaakeaacqWGZbWCdaWgaaWcbaGaemyEaKhabeaakiabdohaZnaaBaaaleaacqWG4baEaeqaaaaaieaakiab+bcaGiab+fgaHjab+5gaUjab+rgaKjabbccaGiab=z9a2jabg2da9maalaaabaGae43Cam3aaSbaaSqaaiabdMha5bqabaaakeaacqGFZbWCdaWgaaWcbaGaemiEaGhabeaaaaGccqGGUaGlaaaa@643F@

The original SAS code for the CCC index is available from [28]. We have adapted the code for S-Plus and R, which is available from the corresponding author.

### Robust statistical test for non-linearity

The relation between microarray and qrPCR FCs was assessed for curvature (Figure 4) by applying the Cramér-von Mises test to residuals generated by the Theil-Sen algorithm [29,30]; see [31] for a description of the procedure and S-Plus functions [see Additional file 1 for more information].

### Empirical study design overview

The biological samples used in the microarray study and the subsequent validation by qrPCR were obtained from three replicate studies. First, the cell culture experiment was performed three distinct times, and every time, the 2 samples (1 control and 1 treatment) were divided into several smaller aliquots. For the microarray hybridization, total RNA was extracted from these smaller aliquots: (1 control + 1 treatment) × 3 experiments = 6 samples. From these total RNA samples, five aliquots of total RNA were labelled and hybridized onto Affymetrix GeneChips; hence 6 samples × 5 aliquots = 30 Genechips. For qrPCR, total RNA was extracted from a second set of the smaller aliquots. For each gene tested, we performed 6 technical replicates of the qrPCR on each of the 6 samples.

### Cell culture, treatment, and RNA extraction

Mouse 3T3-L1 cells, obtained from ATCC, were grown in DMEM (Invitrogen Canada Inc.) containing 10% charcoal/dextran treated fetal bovine serum (Hyclone), 2 μM L-glutamine (Invitrogen) and 100 U/mL penicillin/streptomycin (Invitrogen). Two parallel cultures containing cells seeded at a density of 6 × 10^5 ^per 150-mm plate were grown for 72 h (to confluence). The culture media was replaced and the cells were incubated for an additional 48 h. The cells were then treated with 1 μM dexamethasone (Sigma; dissolved in ethanol) or ethanol (control), for 3 h and were harvested by adding 6 mL Trizol reagent (Invitrogen) directly to each culture dish. The experiment was repeated three times using successive cell passages. Total cellular RNA was prepared according to the manufacturer's instructions. The samples were quantified by spectrophotometry and the RNA integrity was assessed using Agilent BioAnalyser RNA LabChips.

### Microarray probe preparation and hybridization

Biotinylated cRNA probes were prepared for microarray analysis according to the manufacturer's instructions, using 10 μg of total RNA. Five aliquots of each sample were used for probe preparation, and the probes were hybridized overnight to Affymetrix MG-U74Av2 GeneChips (30 in total). Following hybridization, non-specifically bound probe was removed by washing using the GeneChip Fluidics Station 400 (Affymetrix). Specifically bound probe was detected by incubating the arrays with streptavidin phycoerthryin (Molecular Probes) and biotinylated anti-streptavidin antibody (Vector Laboratories) and scanning the chips using a Gene Array Scanner (Agilent). To minimize technical variability, RNA processing steps (RNA extraction, probe labeling and microarray hybridization) were performed in parallel for all samples.

### Microarray data processing

Data for the three experiments were analyzed on a per experiment basis. For each experiment, data were normalized by the robust multi-array average (RMA) algorithm [32]. Differential expression was tested by independent t-tests and corrected for multiple testing using the false discovery rate procedure [FDR, [33]]. Two-hundred forty-two genes were significantly differentially expressed in the same direction in all three experiments (FDR *q *= 0.05). Modified t-tests using the significance analysis of microarrays procedure [SAM, [34]] were also computed, with deltas of 0.25, 0.425, and 0.54269 for experiments 1, 2 and 3, respectively. These deltas corresponded to a false positive rate of approximately 0.05 and yielded 400 genes which were significantly differentially expressed in the same direction in all three experiments (see also the MIAME document [Additional file 9]). The intersection of these two lists contained 241 of the 242 genes identified by t-test alone. Because of the large overlap of the two methods, we selected the list generated by t-test as our final gene list.

These stringent criteria were adopted for two reasons. We wished to strictly minimize the false positive rate so as to reflect as accurately as possible the simulation data while at the same time not using a criterion that was redundant with the validation indices under examination. It is probable that this approach overlooked a number of differentially expressed genes. As such, although it served the methodological purposes of the present study, we do not recommend it as a general analytical strategy inasmuch as the false negative rate was likely unfavorably high.

### Selection of differentially expressed genes for validation

The within-experiment log_2 _FC averages for the 242 consistently statistically significant genes were calculated and then averaged across experiments. Downregulated and four upregulated outlier genes (> 5 MADs) which would have adversely affected statistical analysis were eliminated from the list. Twenty-nine genes from the remaining 150 upregulated genes were selected for validation: the top 15 FCs and one gene randomly selected from each of 15 rank-ordered strata.

### Oligonucleotide primer design for quantitative real time polymerase chain reaction

The cDNA and genomic sequences of all selected genes were obtained and the Primer 3 web tool [35] was used to select pairs of oligonucleotide primers with an optimal melting temperature of 60°C. Primer pairs were selected to span an intron/exon junction, except for a few genes where this was not possible, such as intronless genes.

### Quantitative real time polymerase chain reaction

All primer pairs were tested by the polymerase chain reaction [1× reaction buffer, 3.5 μM MgCl_2_, 0.2 mM dNTP, 0.2 μM of each primer, 5 ng cDNA template (labeled as above), 0.25 U Hotstart Taq polymerase (Qiagen)] and agarose gel electrophoresis to verify the presence of a single band of the predicted size. Selected pairs were tested in quantitative real time polymerase chain reactions (qrPCR) on an ABI Prism^® ^7900HT sequence detection system (Applied Biosystems). Each combination of 50, 150 and 300 nM of forward and reverse primer was tested in duplicate reactions [1× reaction buffer, 3.5 μM MgCl_2_, 0.2 mM dNTP, varying concentrations of primer, 0.04 μM ROX (Molecular probes), 1× SYBR-green 1 (from a 10 000× stock, Molecular probes), 1× bovine serum albumin (New England Biolabs), 5 ng cDNA template, 0.25 U Hotstart Taq polymerase (Qiagen)] and the optimal reaction condition was selected. For each gene, we prepared 6 technical replicate reactions of the following: a calibration curve from a two-fold dilution series ranging from 20 to 0.078 ng of cDNA (9 dilutions), a control containing no cDNA, and each of the six experimental samples; 16 groups in total, for a total of 96 qrPCR reactions. The placement of the 16 groups of qrPCR reactions within the 384-well plates was randomized for every gene such that the samples would not always be located at the same place on the plate. All the qrPCR reactions for a given gene were run in parallel on the same plate. The results from the three control and the three treatment samples were then calculated by comparison to the calibration curve. Seven standard curve data points (for five of the 29 genes) had large influence on the regression slope (as indicated by |standardized slope *dfBeta*| values > 1, [27]). These data points were deleted, and the data were recalibrated. The experimental samples were calibrated according to their respective standard curve equations. Fifty-four of the 1044 calibrated values were found to be outliers (defined as one-and-a-half (1.5) times the inter-quartile range beyond the 25^th ^and 75^th ^percentile values). These outliers were deleted prior to averaging.

## Abbreviations

CCC : concordance correlation coefficient.

FC : fold-change.

FDR : false discovery rate.

qrPCR: quantitative real time polymerase chain reaction.

## Authors' contributions

MM designed, performed, analyzed the qrPCR study and wrote the article. OZW and CM wrote the data processing code and performed the microarray and qrPCR data analysis. AM designed and performed the cell culture experiment. RS designed the microarray experiment and contributed to writing of the manuscript. RN contributed to the conception and design of the study, devised the statistical analysis, wrote and revised the article. All authors read and approved the final manuscript.

## Supplementary Material

Additional File 1 This file provides additional details on particular tests and procedures in a section entitled "Supplementary methods". This file contains supplementary tables 1, 2 and 3, as well as the supplementary bibliography. These tables primarily concern the validation simulations. Also included in this file are the captions for the supplementary figures [Additional files 2, 3,4,5,6,7]. Click here for file

Additional File 2 This file provides supplementary boxplots illustrating the differences between the three microarray experiments with respect to their observed distributions of both FC and variability. Scatterplots are also used to provide a qualitative sense of the precision of expression measurements when challenged with various levels (experimental, biological) of variability. Click here for file

Additional File 3 This file contains figures in the style of figure 4 that pair microarray and qrPCR data across experiments. These figures specifically examine the random-stratified strategy. Click here for file

Additional File 4 Similar to additional file 3, except the "top ranked" strategy is examined instead. Click here for file

Additional File 5 This file presents figure 4 using an alternative FC metric for the qrPCR data. FCs assuming an exact doubling per cycle are substituted in for the standard-curve-based FC estimates used in main manuscript. Click here for file

Additional File 6 This file contains figures illustrating the consequences of excluding low FCs (<0.5 in log space) from figures 4H, 4J, and 4L. Click here for file

Additional File 7 This file presents figure 5 using an alternative FC metric for the qrPCR data. FCs assuming an exact doubling per cycle are substituted in for the standard-curve-based fold-change estimates used in main manuscript. Click here for file

Additional File 8 This Excel spreadsheet contains supplementary table 4. This table provides data (such as FCs, *p*-values) from both the microarray and qrPCR platforms for the genes selected for validation. Click here for file

Additional File 9 MIAME_Description_051008. This pdf document contains the minimum information about microarray experiment description of the microarray experiment used in this paper. Click here for file

## Figures and Tables

**Table 1:** List of genes upregulated in the microarray experiments selected for validation by qrPCR

**Category**	**Affymetrix**	**RefSeq Title**	FC Microarray*				FC PCR*			
	**Probe Set ID**		**Experiment**			**Average**	**Experiment**			**Average**
			**1**	**2**	**3**		**1**	**2**	**3**	
										
**Top 15**										

1	104598_at	dual specificity phosphatase 1	3.96	4.52	5.37	4.58	6.93	6.74	8.36	7.31
2	98817_at	follistatin	3.20	4.43	3.83	3.79	4.88	4.92	3.65	4.44
3	93624_at	RIKEN cDNA 8430417G17	3.49	3.22	4.30	3.64	9.74	10.50	7.16	9.01

4	93319_at	RAS p21 protein activator 3	2.74	5.11	3.13	3.52	4.88	3.34	2.15	3.27
5	160894_at	CCAAT/enhancer binding protein delta	3.29	2.95	3.96	3.37	4.49	6.98	7.42	6.15
6	97844_at	regulator of G-protein signaling 2	2.79	3.83	3.00	3.17	3.65	3.21	2.70	3.16

7	160564_at	lipocalin 2	3.42	2.85	3.27	3.17	8.23	9.81	7.30	8.39
8	96346_at	cysteine dioxygenase 1, cytosolic	2.88	3.15	3.34	3.11	2.52	3.41	2.31	2.71
9	160359_at	RIKEN cDNA 1190002H23	3.06	2.65	3.18	2.95	6.20	6.14	4.23	5.44

10	92276_at	mitogen-activated protein kinase kinase kinase 6	2.75	2.89	3.14	2.92	4.27	4.95	5.42	4.86
11	101030_at	ras homolog gene family, member AB	2.81	2.38	3.77	2.93	4.93	5.82	5.56	5.42
12	102867_at	TEA domain family member 4	2.85	2.70	3.00	2.85	4.38	5.11	4.43	4.63

13	102896_at	downstream of tyrosine kinase 1	2.94	2.27	3.39	2.83	2.55	2.06	2.60	2.39
14	93573_at	metallothionein 1	2.83	2.42	2.94	2.72	3.09	3.14	3.36	3.19
15	97950_at	xanthine dehydrogenase	2.11	3.89	2.24	2.64	4.64	4.82	2.49	3.82

										
**Stratified**										

1	160894_at	CCAAT/enhancer binding protein delta	3.29	2.95	3.96	3.37	4.49	6.98	7.42	6.15
2	102955_at	nuclear factor, interleukin 3 regulated	2.59	2.43	3.06	2.68	4.02	3.42	3.12	3.50
3	92777_at	cysteine rich protein 61	2.44	2.20	2.65	2.42	2.74	1.77	2.55	2.31

4	99027_at	Bcl2-like	2.21	1.56	2.49	2.05	2.62	1.85	2.19	2.20
5	103644_at	dipeptidase 1 (renal)	1.89	2.04	1.87	1.93	5.09	4.49	2.63	3.92
6	100952_at	stromal interaction molecule 1	1.81	1.57	1.99	1.78	2.00	1.87	**1.26**	1.68

7	102984_g_at	MAD homolog 1	1.56	1.68	2.07	1.75	1.23	**0.95**	**1.15**	1.11
8	161228_f_at	enabled homolog	1.69	1.61	1.57	1.62	1.68	1.43	1.42	1.51
9	103432_at	interferon-stimulated protein	1.63	1.31	1.65	1.52	1.59	1.40	1.40	1.46

10	96106_at	RIKEN cDNA 2400006P09	1.41	1.28	1.79	1.48	1.56	1.50	1.21	1.42
11	100019_at	chondroitin sulfate proteoglycan 2	1.32	1.65	1.37	1.44	1.61	1.58	**1.04**	1.38
12	160924_at	cDNA sequence BC028953	1.31	1.40	1.39	1.36	1.62	2.03	**1.13**	1.55

13	96183_at	forkhead box P1	1.34	1.28	1.31	1.31	2.06	1.68	1.52	1.74
14	103890_at	expressed sequence AW538196	1.15	1.30	1.29	1.24	1.72	**0.82**	**2.00**	1.41
15	104268_at	interleukin 6 receptor, alpha	1.18	1.20	1.19	1.19	2.75	2.08	1.48	2.04

* FC is the anti-log of the average log_2 _FC across the three experiments; for the microarray data, a weighted (by sample size) average was calculated (n = 5 for Experiments 1 and 2 and n = 4 for Experiment 3). All microarray FCs were significant (FDR q = 0.05); all non-bolded PCR Fcs were significant (nominal *p *< .05).
