# Supplementary material for: A methodology for global validation of microarray experiments
Source: BMC Bioinformatics. 2006 Jul 5;7:333. doi: 10.1186/1471-2105-7-333 (PMC1539027; doi:10.1186/1471-2105-7-333)
Supplement: Additional File 1 — This file provides additional details on particular tests and procedures in a section entitled "Supplementary methods". This file contains supplementary tables 1, 2 and 3, as well as the supplementary bibliography. These tables primarily concern the validation simulations. Also included in this file are the captions for the supplementary figures [Additional files 2, 3,4,5,6,7]. [file 1471-2105-7-333-S1.pdf]

Supplementary methods

Robust statistical test for non-linearity

Three data points (red data points in Figures 4B, J, and L) had large influence on the regression slope (as indicated by |standardized slope *dfBeta*| values > 1 [1]). Deleting these values, however, did not materially change any of the index values (data not shown).

Also, the loess fits to the data suggest a non-linear relationship between microarray and qrPCR FCs for the stratified sampling results (yellow lines in Figures 4H, J, and L), raising the issue whether low FCs ($\log_2 < 0.5$) observed in the microarray data were validated by qrPCR. We conducted two tests of non-linearity to investigate this issue further. OLS tests for quadratic effects yielded inconsistent results; *p* values for the quadratic term for Experiments 1-3 were 0.28, 0.08, and 0.004, respectively. Results of a robust regression approach for detecting curvature were likewise inconsistent, yielding $p > 0.10$ for Experiments 1 and 2 and $p < 0.10$ for Experiment 3 (Cramér-von Mises test applied to residuals generated by the Theil-Sen algorithm [2, 3]; see [4] for a description of the procedure and S-Plus functions). We also re-calculated the various indices for the random-stratified data within the graphically-estimated linear range only. Additional file 6 presents the values for the various measures with microarray $\log_2$ FCs < 0.5 removed (red data points in Additional file 6, panels A-C). Although precision increased and accuracy decreased, the CCC estimate did not change materially. Moreover, the marked

superiority of random-stratified sampling relative to top-ranked sampling was maintained.

Processing with additional algorithms and comparison with qrPCR results

Dchip (without and with mismatches subtraction), PLIER, GC-RMA and MAS5.0 were applied to the microarray data and FC estimates were calculated for each of the 3 experiments. For each method, a weighted-average FC value of the 3 experiments was calculated. The validation indices were calculated by comparing the average microarray FC of the 29 genes selected for validation to their corresponding qrPCR FC estimates. Outlier points were detected by determining if their |standardized slope *dfBeta*| values > 1 [1], and deleted accordingly.

Supplementary Figure Legends

Additional file 2

Boxplots of within-experiment variability (t-test denominator) (A) show that Experiment 1 displays an overall lower variability than experiments 2 and 3. Boxplots of effect sizes (the fold-change values, which are the t-test numerator) (B, and zoomed-in in C) show that overall the FCs are larger in Experiment 1 than in Experiments 2 and 3. Combined together, these effects explain why Experiment 1 displayed so many significant genes. The scatter plots of intensity measurement (RMA expression estimates) (D-E) display high precision (Pearson *r*).

Additional file 3 and 4 – Comparisons of biological validation samples for validation of microarray experiment by qrPCR.

As Figure 4, except that comparison of the $\log_2$ fold-change (FC) values for microarrays and qrPCR were performed between biological replicates (between experiments). Concordance between microarray and qrPCR FCs (averaged across within-experiment replicates) for random-stratified sampling (Additional file 3) and top-ranked (Additional file 4) methods for each microarray experiment is shown in the six scatter plots on the right and the corresponding MvA plots are on the left. Validation using the random-stratified sampling approach consistently yields better results than validation using the top-ranked approach. The regression lines are: the identity line (red), least-squares (blue) and loess (yellow).

Additional file 5 - Validation of microarray experiment by qrPCR.

As Figure 4, except that the $\log_2$ fold-change (FC) values for qrPCR were determined assuming a linear two-fold amplification for each cycle of the PCR reaction. Concordance between microarray and qrPCR FCs (averaged across within-experiment replicates) for top-ranked ($n = 15$) and random-stratified sampling methods ($n = 15$) for each microarray experiment is shown in the six scatter plots on the right (B, D, F, H, J, L); corresponding MvA plots are shown on the left (A, C, E, G, I, K). Validation using the random-stratified sampling approach consistently yields better results than validation using the top-ranked approach. The regression lines are: the identity line (red), least-squares (blue) and loess (yellow).

Additional file 6 - Validation of microarray experiments by qrPCR (low FCs removed).

Same data as in Figure 4H, J, and L but with log_2 FCs < 0.5 (red +’s) removed. The regression lines are: the identity line (red) and least-squares (blue).

Additional file 7 - Validation of microarray experiments by qrPCR (data averaged across experiments).

As Figure 5, except that the log_2 fold-change (FC) values for qrPCR were determined assuming a linear two-fold amplification for each cycle of the PCR reaction. Data in (A) were averaged across Additional file 5B, D, and F; data in (B) were averaged across Additional file 5H, J, and L. The regression lines are: the identity line (red), least-squares (blue) and loess (yellow).

Supplementary Table 1. Validation indices for the benchmark and the sampling strategy samples.

Slope

	Mean	SD	Median	IQR[1]	No. of Outliers[2]
Benchmark	0.80	0.06	0.80	0.06	16
Random-stratified	0.80	0.20	0.80	0.26	8
Random	0.80	0.23	0.80	0.29	22
Top	0.80	0.57	0.81	0.66	28

Y-intercept

	Mean	Standard Deviation	Median	IQR[1]	No. of Outliers[2]
Benchmark	0.20	0.06	0.20	0.06	13
Random-stratified	0.21	0.21	0.21	0.26	11
Random	0.20	0.23	0.19	0.28	30
Top	0.20	0.78	0.19	0.91	29

Precision

	Mean	Standard Deviation	Median	IQR[1]	No. of Outliers[2]
Benchmark	0.80	0.04	0.80	0.05	21
Random-stratified	0.81	0.11	0.83	0.13	34
Random	0.78	0.16	0.82	0.17	47
Top	0.46	0.27	0.51	0.36	16

Accuracy

	Mean	Standard Deviation	Median	IQR[1]	No. of Outliers[2]
Benchmark	1.00	0.004	1.00	0.004	59
Random-stratified	0.96	0.04	0.98	0.04	56
Random	0.95	0.05	0.97	0.05	68
Top	0.67	0.17	0.69	0.25	2

CCC

	Mean	Standard Deviation	Median	IQR[1]	No. of Outliers[2]
Benchmark	0.79	0.04	0.80	0.05	21
Random-stratified	0.78	0.12	0.81	0.14	31
Random	0.75	0.16	0.78	0.18	46
Top	0.32	0.22	0.33	0.30	7

[1] IQR = inter-quartile range (dispersion of middle 50% of the data)
[2] Outlier thresholds are defined as (25th percentile - 1.5 x IQR) and (75th percentile + 1.5 x IQR). They are shown as open circles beyond the hinges of the boxplots in Figure 1C-G in the main article.

Supplementary Table 2. Differences on validation indices between sampling strategy and benchmark samples.

Slope

	Mean	SD	Median	IQR[1]	No. of Outliers[2]
Random-stratified	0.00	0.19	-0.01	0.25	7
Random	0.00	0.22	0.01	0.27	23
Top	0.01	0.56	0.00	0.65	30

Y-intercept

	Mean	Standard Deviation	Median	IQR[1]	No. of Outliers[2]
Random-stratified	0.00	0.19	0.00	0.26	6
Random	0.00	0.22	-0.01	0.27	26
Top	-0.01	0.78	0.00	0.91	29

Precision

	Mean	Standard Deviation	Median	IQR[1]	No. of Outliers[2]
Random-stratified	0.01	0.11	0.03	0.12	27
Random	-0.02	0.15	0.02	0.16	47
Top	-0.34	0.27	-0.29	0.36	19

Accuracy

	Mean	Standard Deviation	Median	IQR[1]	No. of Outliers[2]
Random-stratified	-0.03	0.04	-0.02	0.04	57
Random	-0.04	0.05	-0.03	0.05	67
Top	-0.33	0.17	-0.31	0.25	1

CCC

	Mean	Standard Deviation	Median	IQR[1]	No. of Outliers[2]
Random-stratified	-0.01	0.11	0.01	0.13	25
Random	-0.05	0.16	-0.01	0.17	43
Top	-0.47	0.21	-0.47	0.29	6

[1] IQR = inter-quartile range (dispersion of middle 50% of the data)
[2] Outlier thresholds are defined as (25th percentile - 1.5 x IQR) and (75th percentile + 1.5 x IQR). They are shown as open circles beyond the hinges of the boxplots in Figure 2C-G in the main article.

Estimates of the accuracy coefficient were slightly lower than the benchmark samples for random-stratified and random samples (mean differences of -0.03 and -0.04, respectively). Precision estimates were slightly lower for random sampling (mean difference = -0.02), while random-stratified sampling produced slightly upwardly biased precision estimates (mean difference = 0.01). The extent of the bias in the precision estimates of both random and random-stratified sampling will vary with the true population correlation and with sample size. This bias should be, however, negligible in microarray validation studies.

For random sampling, there are formulas which provide approximate corrections for the negative bias, which can be as high as 0.03-0.04 (Zimmerman et al., 2003). To estimate the size of the bias for the type of stratification in the present study, we conducted additional simulations (10,000 runs at a time) in which we

varied the number of "genes" selected per stratum (1-9 of 10). For randomly stratified data, the upward bias (mean difference with the benchmark data) of the sample correlation coefficient ranged from a high of .0040 (n = 1 per stratum) to a low of 0.0003 (n = 9 per stratum) [5].

Supplementary Table 3. Validation indexes from comparisons of FC estimates of qrPCR and selected algorithms.

Algorithm	CCC	Precision	Accuracy	Y-intercept	Slope	ICC
Dchip random-stratified	0.51	0.58	0.88	0.39	0.72	0.59
Dchip top-ranked	0.05	0.19	0.28	1.37	0.52	-0.53
Dchip (-MM) random-stratified	0.51	0.51	0.98	0.34	0.61	0.58
Dchip (-MM) top-ranked	0.25	0.41	0.62	0.69	0.80	-0.19
PLIER top random-stratified	0.76	0.85	0.89	-0.16	1.33	0.66
PLIER top top-ranked	0.43	0.73	0.59	0.04	1.24	0.34
GC-RMA random-stratified	0.60	0.74	0.81	-0.03	0.73	0.65
GC-RMA top-ranked	0.48	0.57	0.85	0.63	0.63	0.47
MAS 5.0 random-stratified	0.76	0.86	0.89	-0.20	0.97	0.78
MAS 5.0 top-ranked	0.69	0.73	0.95	0.16	0.96	0.71

Calculation of the validation indexes after reprocessing of the microarray data with additional algorithms [Dchip (without and with MM subtraction), PLIER, GC-RMA, MAS5.0] shows their relative performance compared to qrPCR.

References

1. Cohen J, Cohen P, West SG, Aiken L: **Applied multiple regression/correlation analysis for the behavioral sciences**, 3rd edn. Mahwah, N.J. ;, London :: L. Erlbaum Associates; 2003.
2. Sen PK: **Estimates of regression coefficient based on Kendall's tau**. *Journal of the American Statistical Association* 1968, **63**(324):1379-&.
3. Theil H: **A rank-invariant method of linear and polynomial regression analysis**. *Indagationes Mathematicae* 1950, **12**:85-91.
4. Wilcox RR: **Applying contemporary statistical techniques**. In. Amsterdam ;, Boston :: Academic Press; 2003: pp. 477-479 and 531-533.
5. Zimmerman DW, Zumbo BD, Williams RH: **Bias in estimation and hypothesis testing of correlation**. *Psicológica* 2003, **24**:133-158.
