# Supplementary material for: A methodology for global validation of microarray experiments
Source: BMC Bioinformatics. 2006 Jul 5;7:333. doi: 10.1186/1471-2105-7-333 (PMC1539027; doi:10.1186/1471-2105-7-333)
Supplement: Additional File 2 — This file provides supplementary boxplots illustrating the differences between the three microarray experiments with respect to their observed distributions of both FC and variability. Scatterplots are also used to provide a qualitative sense of the precision of expression measurements when challenged with various levels (experimental, biological) of variability. [file 1471-2105-7-333-S2.pdf]

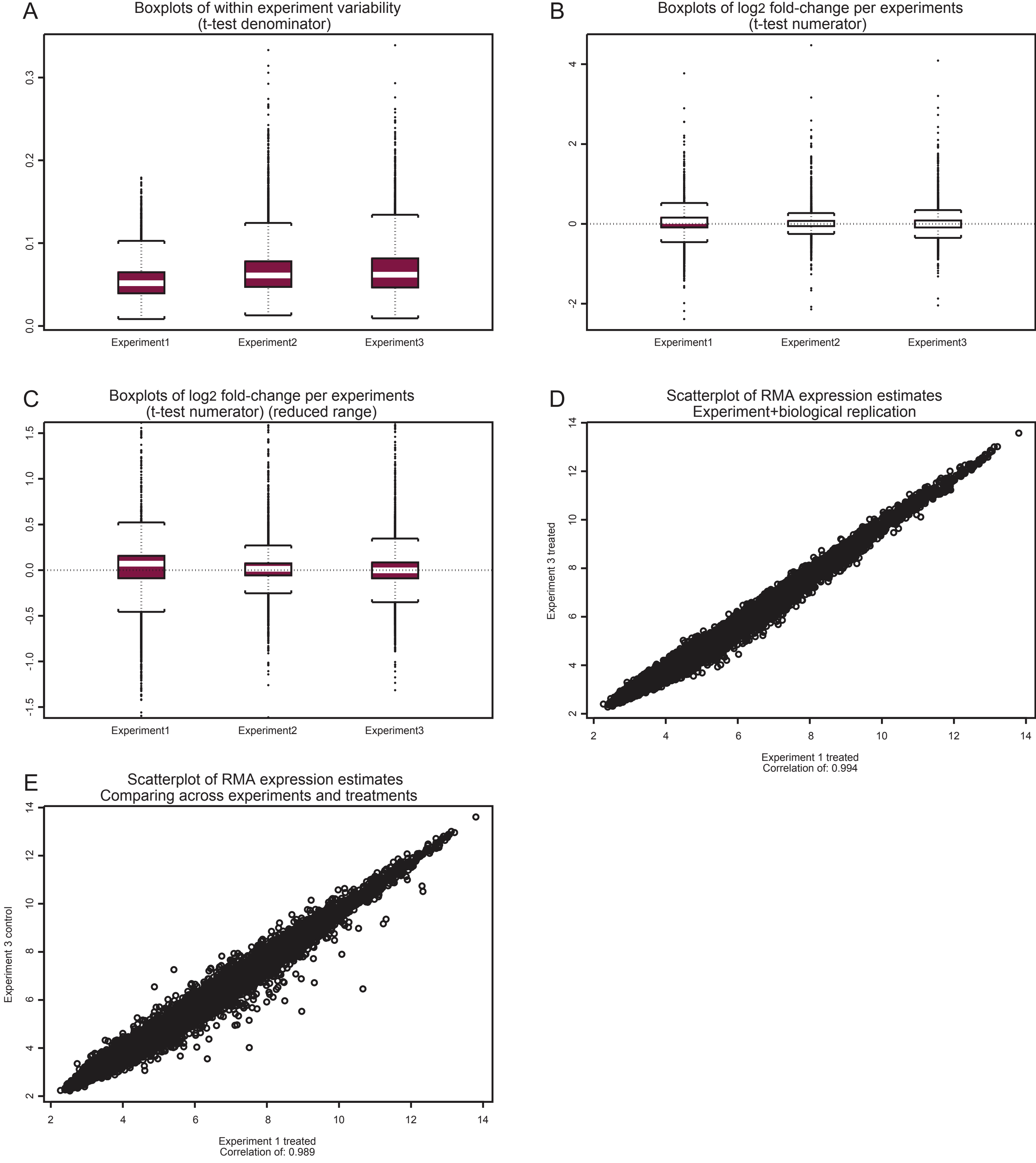

A
Boxplots of within experiment variability
(t-test denominator)
0.0
0.1
0.2
0.3
Experiment1
Experiment2
Experiment3
B
Boxplots of log2 fold-change per experiments
(t-test numerator)
-2
0
2
4
Experiment1
Experiment2
Experiment3
C
Boxplots of log2 fold-change per experiments
(t-test numerator) (reduced range)
-1.5
-1.0
-0.5
0.0
0.5
1.0
1.5
Experiment1
Experiment2
Experiment3
D
Scatterplot of RMA expression estimates
Experiment+biological replication
Experiment 3 treated
2
4
6
8
10
12
14
Experiment 1 treated
Correlation of: 0.994
E
Scatterplot of RMA expression estimates
Comparing across experiments and treatments
Experiment 3 control
2
4
6
8
10
12
14
Experiment 1 treated
Correlation of: 0.989
