# Supplementary material for: A methodology for global validation of microarray experiments
Source: BMC Bioinformatics. 2006 Jul 5;7:333. doi: 10.1186/1471-2105-7-333 (PMC1539027; doi:10.1186/1471-2105-7-333)
Supplement: Additional File 3 — This file contains figures in the style of figure 4 that pair microarray and qrPCR data across experiments. These figures specifically examine the random-stratified strategy. [file 1471-2105-7-333-S3.pdf]

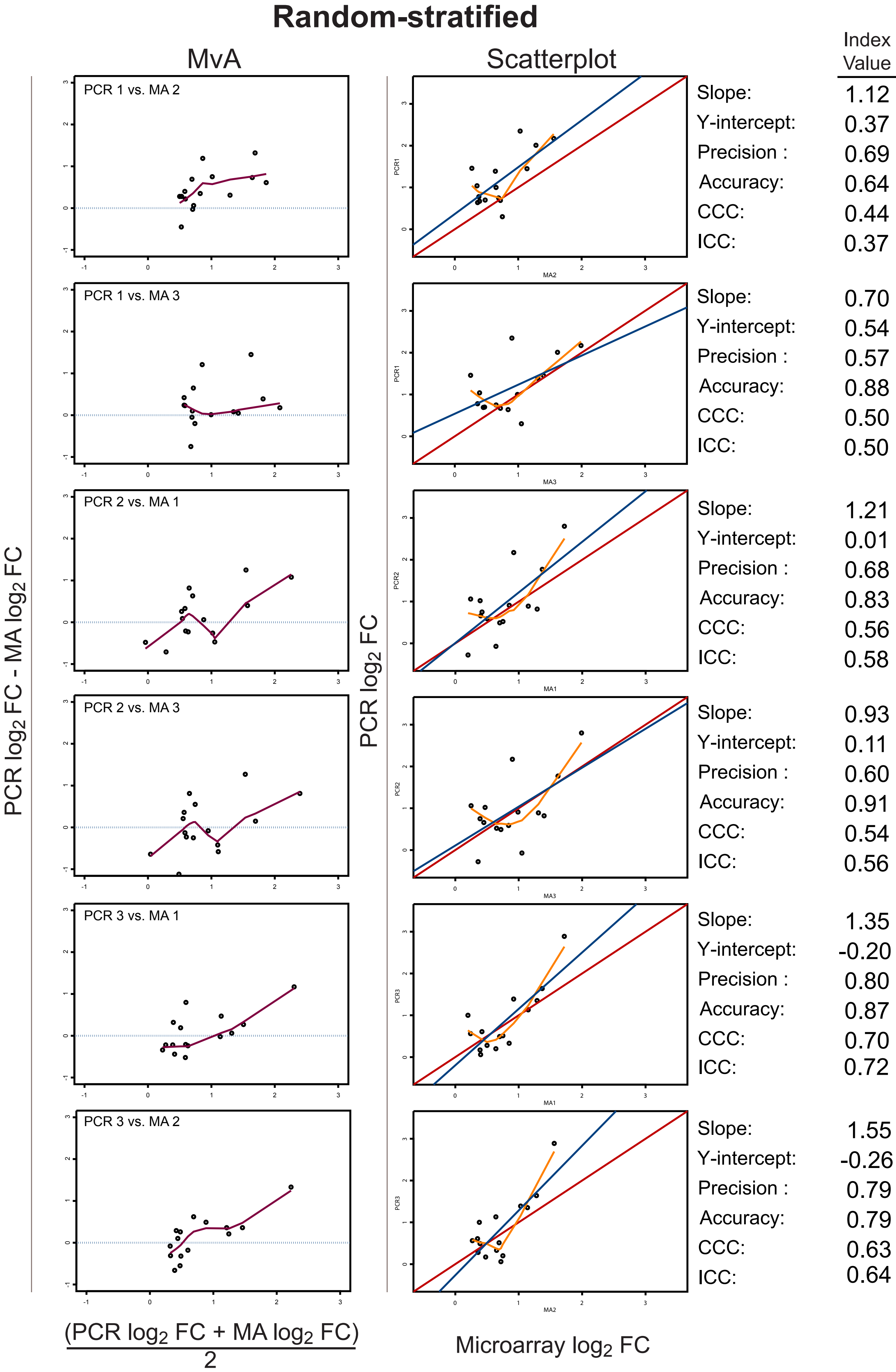
Random-stratified
MvA
Scatterplot
Index Value
PCR 1 vs. MA 2
Slope: 1.12
Y-intercept: 0.37
Precision : 0.69
Accuracy: 0.64
CCC: 0.44
ICC: 0.37
PCR 1 vs. MA 3
Slope: 0.70
Y-intercept: 0.54
Precision : 0.57
Accuracy: 0.88
CCC: 0.50
ICC: 0.50
PCR 2 vs. MA 1
Slope: 1.21
Y-intercept: 0.01
Precision : 0.68
Accuracy: 0.83
CCC: 0.56
ICC: 0.58
PCR 2 vs. MA 3
Slope: 0.93
Y-intercept: 0.11
Precision : 0.60
Accuracy: 0.91
CCC: 0.54
ICC: 0.56
PCR 3 vs. MA 1
Slope: 1.35
Y-intercept: -0.20
Precision : 0.80
Accuracy: 0.87
CCC: 0.70
ICC: 0.72
PCR 3 vs. MA 2
Slope: 1.55
Y-intercept: -0.26
Precision : 0.79
Accuracy: 0.79
CCC: 0.63
ICC: 0.64
PCR log2 FC - MA log2 FC
(PCR log2 FC + MA log2 FC) / 2
PCR log2 FC
Microarray log2 FC
