# Supplementary material for: A methodology for global validation of microarray experiments
Source: BMC Bioinformatics. 2006 Jul 5;7:333. doi: 10.1186/1471-2105-7-333 (PMC1539027; doi:10.1186/1471-2105-7-333)
Supplement: Additional File 4 — Similar to additional file 3, except the "top ranked" strategy is examined instead. [file 1471-2105-7-333-S4.pdf]

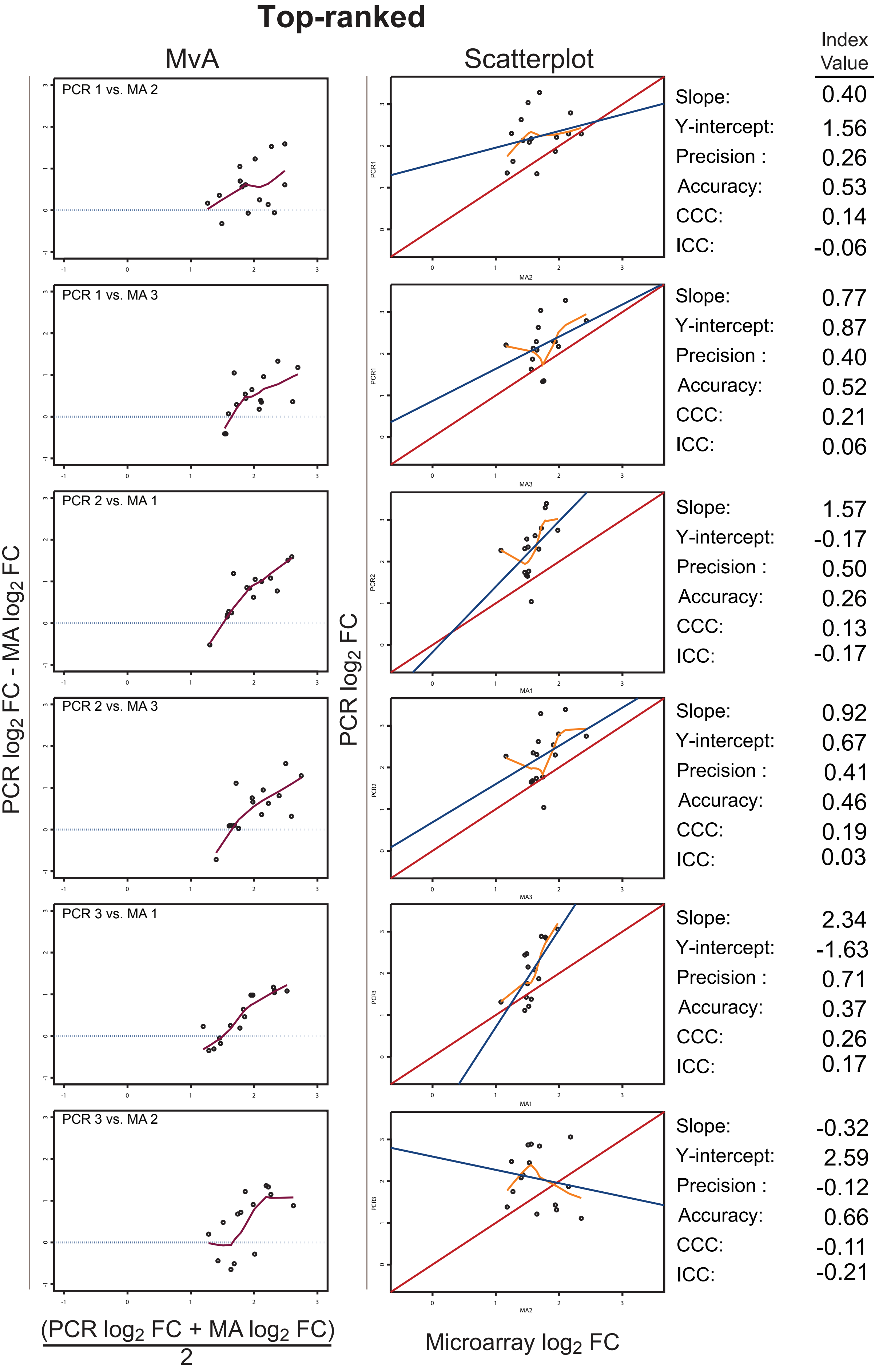

Top-ranked
MvA
Scatterplot
Index Value
PCR 1 vs. MA 2
Slope: 0.40
Y-intercept: 1.56
Precision : 0.26
Accuracy: 0.53
CCC: 0.14
ICC: -0.06
PCR 1 vs. MA 3
Slope: 0.77
Y-intercept: 0.87
Precision : 0.40
Accuracy: 0.52
CCC: 0.21
ICC: 0.06
PCR 2 vs. MA 1
Slope: 1.57
Y-intercept: -0.17
Precision : 0.50
Accuracy: 0.26
CCC: 0.13
ICC: -0.17
PCR 2 vs. MA 3
Slope: 0.92
Y-intercept: 0.67
Precision : 0.41
Accuracy: 0.46
CCC: 0.19
ICC: 0.03
PCR 3 vs. MA 1
Slope: 2.34
Y-intercept: -1.63
Precision : 0.71
Accuracy: 0.37
CCC: 0.26
ICC: 0.17
PCR 3 vs. MA 2
Slope: -0.32
Y-intercept: 2.59
Precision : -0.12
Accuracy: 0.66
CCC: -0.11
ICC: -0.21
PCR log2 FC - MA log2 FC
(PCR log2 FC + MA log2 FC) / 2
PCR log2 FC
Microarray log2 FC
