# Supplementary figures and images for: A methodology for global validation of microarray experiments
Source: BMC Bioinformatics. 2006 Jul 5;7:333. doi: 10.1186/1471-2105-7-333 (PMC1539027; doi:10.1186/1471-2105-7-333)

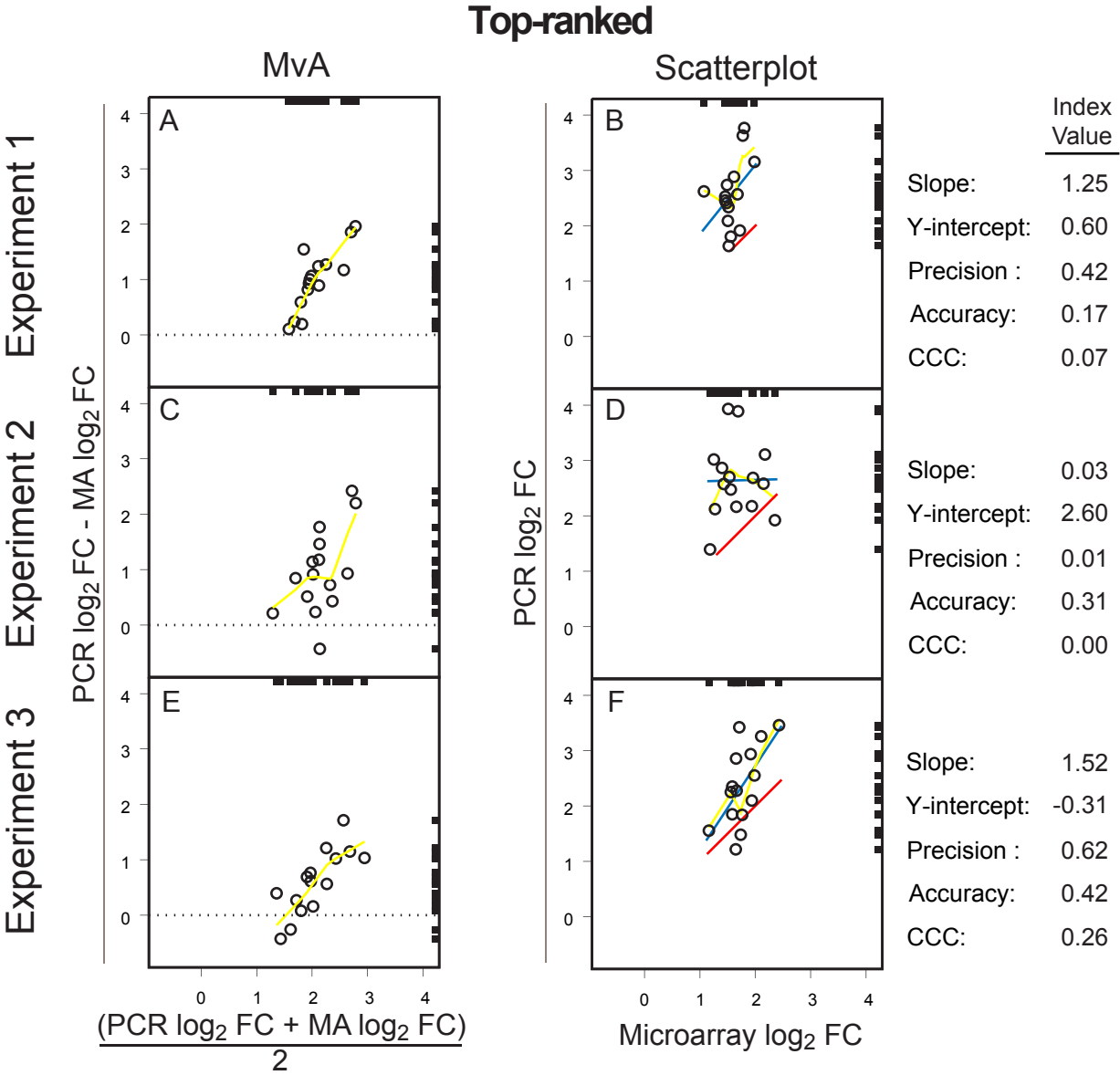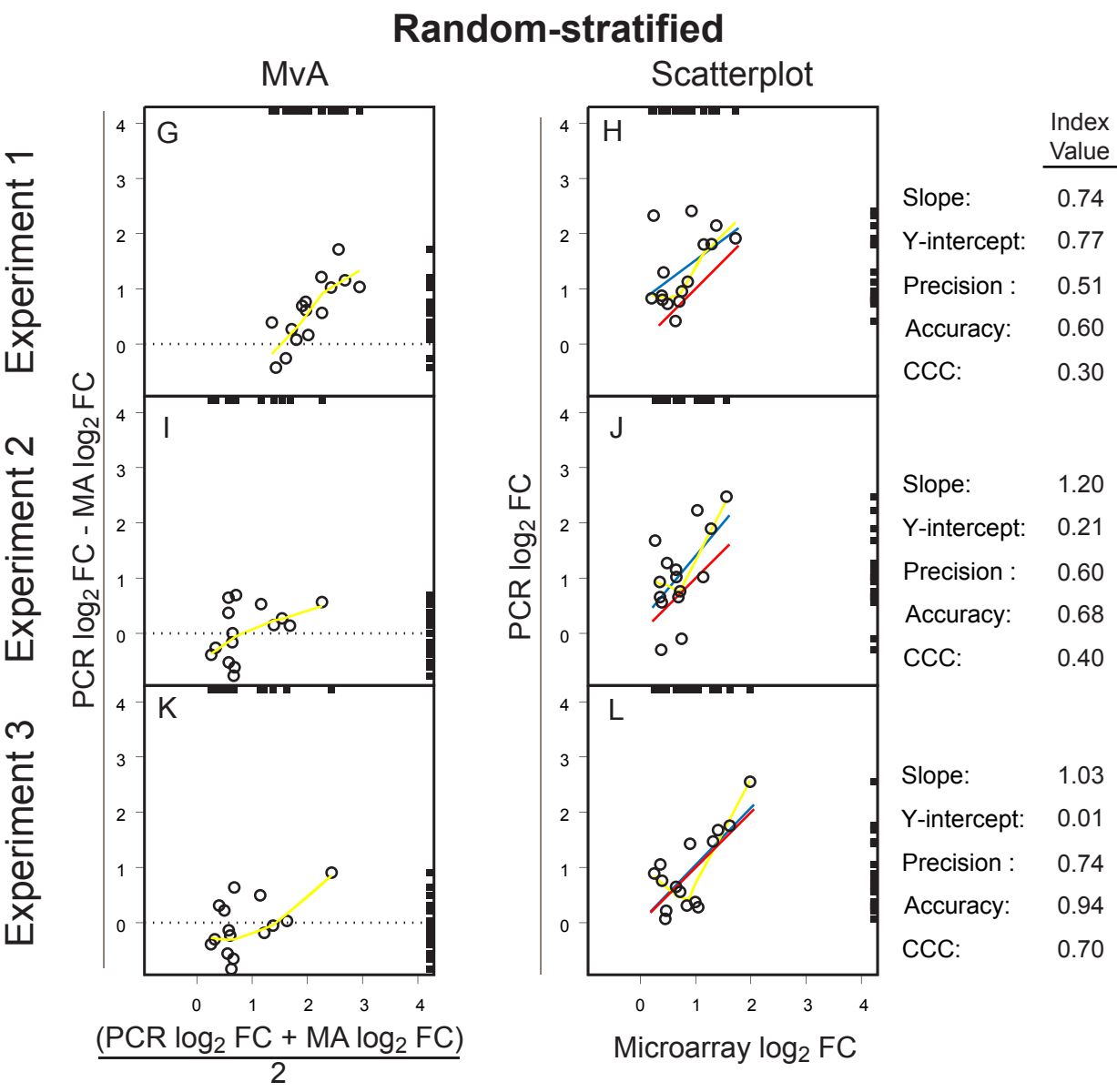

Supplement: Additional File 5 — This file presents figure 4 using an alternative FC metric for the qrPCR data. FCs assuming an exact doubling per cycle are substituted in for the standard-curve-based FC estimates used in main manuscript. [file 1471-2105-7-333-S5.pdf]
