# Supplementary material for: A methodology for global validation of microarray experiments
Source: BMC Bioinformatics. 2006 Jul 5;7:333. doi: 10.1186/1471-2105-7-333 (PMC1539027; doi:10.1186/1471-2105-7-333)
Supplement: Additional File 6 — This file contains figures illustrating the consequences of excluding low FCs (<0.5 in log space) from figures 4H, 4J, and 4L. [file 1471-2105-7-333-S6.pdf]

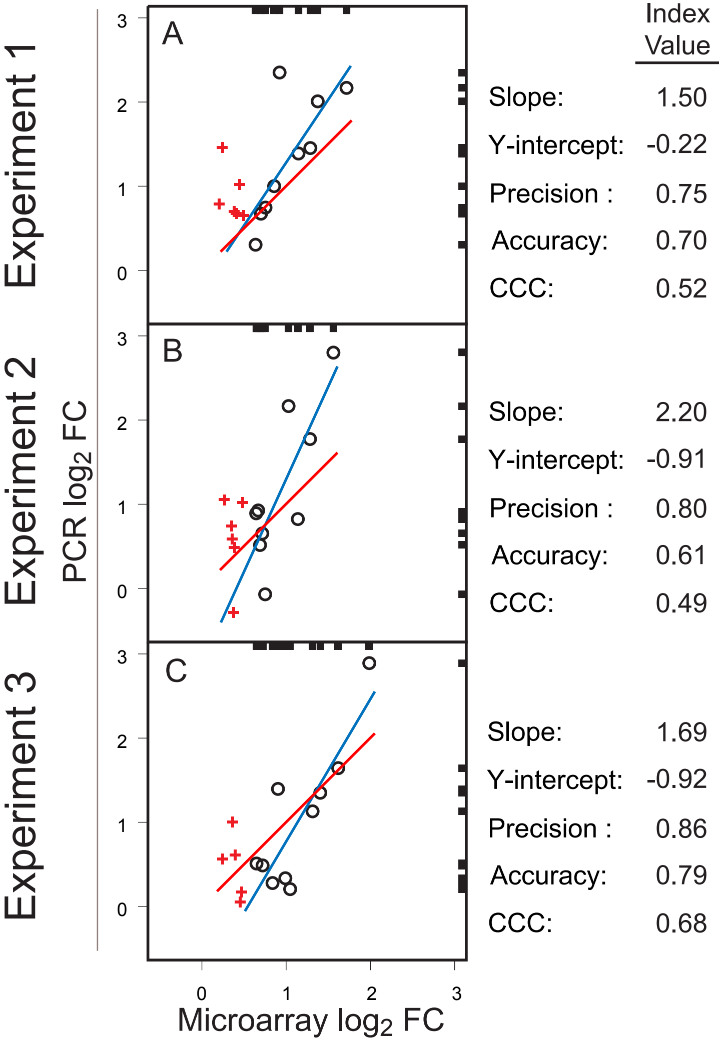
Experiment 1
Experiment 2
Experiment 3
PCR log2 FC
Microarray log2 FC
A
B
C
0
1
2
3
Index Value
Slope: 1.50
Y-intercept: -0.22
Precision : 0.75
Accuracy: 0.70
CCC: 0.52
Slope: 2.20
Y-intercept: -0.91
Precision : 0.80
Accuracy: 0.61
CCC: 0.49
Slope: 1.69
Y-intercept: -0.92
Precision : 0.86
Accuracy: 0.79
CCC: 0.68
