# Supplementary material for: A methodology for global validation of microarray experiments
Source: BMC Bioinformatics. 2006 Jul 5;7:333. doi: 10.1186/1471-2105-7-333 (PMC1539027; doi:10.1186/1471-2105-7-333)
Supplement: Additional File 7 — This file presents figure 5 using an alternative FC metric for the qrPCR data. FCs assuming an exact doubling per cycle are substituted in for the standard-curve-based fold-change estimates used in main manuscript. [file 1471-2105-7-333-S7.pdf]

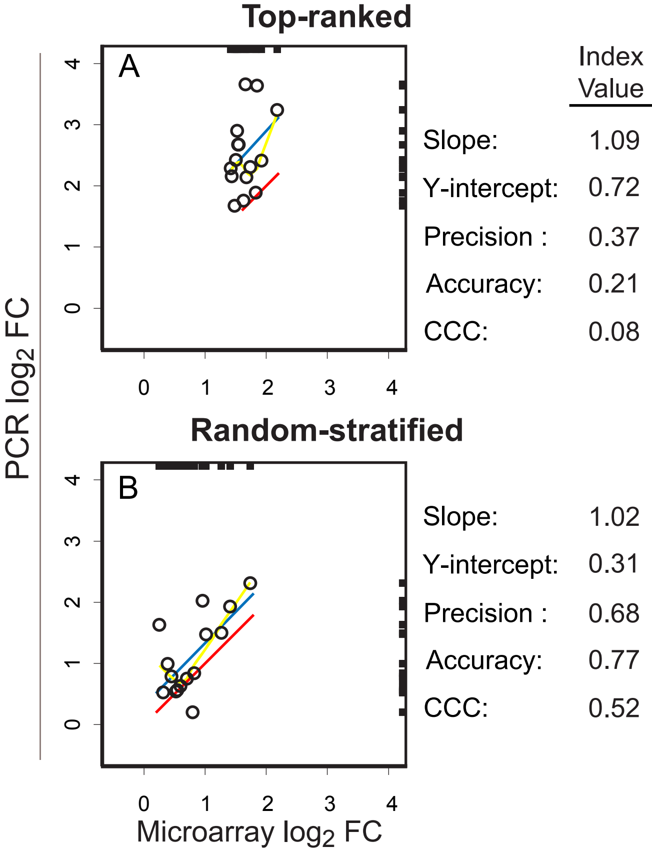

Top-ranked
A
Index Value
Slope: 1.09
Y-intercept: 0.72
Precision : 0.37
Accuracy: 0.21
CCC: 0.08
Random-stratified
B
Slope: 1.02
Y-intercept: 0.31
Precision : 0.68
Accuracy: 0.77
CCC: 0.52
PCR log2 FC
Microarray log2 FC
0
1
2
3
4
