# Supplementary material for: A methodology for global validation of microarray experiments
Source: BMC Bioinformatics. 2006 Jul 5;7:333. doi: 10.1186/1471-2105-7-333 (PMC1539027; doi:10.1186/1471-2105-7-333)
Supplement: Additional File 9 — MIAME_Description_051008. This pdf document contains the minimum information about microarray experiment description of the microarray experiment used in this paper. [file 1471-2105-7-333-S9.pdf]

<u>Description of the Microarray Experiments (Miron et al.).</u>

1. **Experiment Design:**

- **Goal:** To develop a methodology for global validation of microarray experiments

- **Brief description of the experiment:** Validation of microarray findings by lower throughput methods is becoming a requirement for publication in leading scientific journals. We distinguish between validating individual genes and validating entire microarray experiments. We illustrate why the popular strategy of selecting only the most differentially expressed genes for validation generally fails as a global validation strategy and propose random-stratified sampling as a better gene selection method. We also illustrate shortcomings of often-used validation indices such as overlap of significant effects and the correlation coefficient and recommend the concordance correlation coefficient (CCC) as an alternative. We provide recommendations that will enhance validity checks of microarray experiments while minimizing the need to run a large number of labour-intensive individual validation assays.

- **Keywords:** Dose- response, Replicated study

- **Experimental factors:** Treatment with Dexamethasone 1 μM; Replication effects resulting from plate-to- plate and passage-to-passage variation

- **Experimental design:**

Number	Array	Experiment	Group	
1	YF030820MGA01	Exp1	Control	
2	YF030820MGA02	Exp1	Control	
3	YF030820MGA03	Exp1	Control	
4	YF030820MGA04	Exp1	Control	
5	YF030820MGA05	Exp1	Control	
6	YF030820MGA06	Exp2	Control	
7	YF030820MGA07	Exp2	Control	
8	YF030820MGA08	Exp2	Control	
9	YF030820MGA09	Exp2	Control	
10	YF030820MGA10	Exp2	Control	
11	YF030820MGA11	Exp3	Control	
12	YF030820MGA12	Exp3	Control	
13	YF030820MGA13	Exp3	Control	
14	YF030820MGA14	Exp3	Control	
15	YF030820MGA15	Exp3	Control	deleted
16	YF030820MGA16	Exp1	Treatment	
17	YF030820MGA17	Exp1	Treatment	
18	YF030820MGA18	Exp1	Treatment	
19	YF030820MGA19	Exp1	Treatment	
20	YF030820MGA20	Exp1	Treatment	
21	YF030820MGA21	Exp2	Treatment	
22	YF030820MGA22	Exp2	Treatment	
23	YF030820MGA23	Exp2	Treatment	
24	YF030820MGA24	Exp2	Treatment	
25	YF030820MGA25	Exp2	Treatment	
26	YF030820MGA26	Exp3	Treatment	
27	YF030820MGA27	Exp3	Treatment	
28	YF030820MGA28	Exp3	Treatment	
29	YF030820MGA29	Exp3	Treatment	deleted
30	YF030820MGA30	Exp3	Treatment	

- **Quality control steps:** Replicates were obtained to study plate-to-plate variation (5 replicates) and cell passage number (3 replicates)

2. **Samples:**

- **Origin of biological samples:** Murine NIH 3T3-L1 cells were obtained from American Type Culture Collection (Manassas, VA).

- **Manipulation of biological samples:** Early passage mouse NIH 3T3-L1 cells were grown in DMEM medium (Invitrogen Canada Inc.) containing 10% charcoal/dextran treated fetal bovine serum (Hyclone), 2 mM L-glutamine (Invitrogen Canada Inc.) and 100 U/mL penicillin/streptomycin (Invitrogen Canada Inc.). Cells ($6x10^5$) were grown in 150-mm plates for a period of 72h (to confluence); the media was replaced with fresh media and the cells were incubated for an additional 48h. Next, the cells were treated with 1 μM dexamethasone (Sigma; dissolved in ethanol) or ethanol (control), for 3h. The

cells were harvested at the indicated time by removing the culture media and adding Trizol reagent (6mL per 150-mm plate)(Invitrogen Canada Inc.) directly to the culture dish. The sample was next collected into 1.5 mL tubes and frozen immediately. The experiment was repeated three times, on different days. Then, to minimize technical variability, RNA processing steps (RNA extraction, probe labeling and microarray hybridization) were performed in parallel for all samples. Total cellular RNA was prepared according to the manufacturer's instructions. The samples were quantified by spectrophotometry and the RNA integrity was assessed using Agilent BioAnalyser RNA LabChips.

- **Experimental factor value for each experimental factor:**
  - i. Dexamethasone: None or 1 μM
  - ii. Passage number: First, Second or Third
  - iii. Plate replicate: 1, 2,3,4,or 5

- **Probe labeling:** To prepare probes for microarray analysis for each sample, five independent reactions of 10 μg of total RNA was dissolved in 10 μL of DEPC-treated water and 1 μL (100 pmol) of T7- (T)24 primer (Genosys, GGCCAGTGAATTGTAATACGACTCACTATAGGGAGGCGG- (T)24) was added. The primer-RNA mixture was denatured for 10 minutes at 70C, and then chilled on ice. First strand cDNA synthesis was performed using 2 μL of Superscript II reverse transcriptase (Invitrogen Canada Inc.) in a 20 mL reaction volume containing 10 μM DTT, 500 μM each dNTP and 1x First Strand Buffer (all Invitrogen Canada Inc.) for 60 minutes at 42C. Second strand synthesis was performed by adding 40U DNA Polymerase I, 10U E. Coli DNA ligase, 2U RNAse H in a final reaction volume of 150 μL containing 1x Second Strand Buffer (all Invitrogen Canada Inc.). The reaction was incubated at 16C for two hours and stopped by adding 10 μL of 0.5 M EDTA (Sigma). Following second strand synthesis, the probe cDNA was purified by phenol chloroform extraction using Phase-Lock tubes (5'-3') and redissolved in 20 μL of DEPC-treated water. Biotinylated probe was prepared from the entire cDNA reaction using the ENZO Bioarray High Yield RNA Transcript Labeling Kit (ENZO diagnostics). The probe synthesis reaction was performed at 37C for 5 hours with occasional agitation. The biotinylated probe was purified using an RNeasy spin column (Qiagen), eluted in 80 μL of DEPC-treated water, quantified by spectrophotometry and probe quality was assessed using Agilent BioAnalyser RNA LabChips. The average probe length was reduced by incubating the purified probe in 1x Fragmentation Buffer for 35 minutes at 95C.

- **External controls:** Affymetrix Control Oligonucleotide B2

3. **Hybridization procedures and parameters:**

- **Microarray hybridization:** The hybridization mixture was prepared by mixing 15 mg of biotinylated probe with Control Oligonucleotide B2 (final concentration 50 pM) (Affymetrix), Herring Sperm DNA (final concentration 0.1 mg/ml) (Research Genetics), Acetylated BSA (final concentration 0.5 mg/ml) (Invitrogen Canada Inc.) in a final volume of 300 mL of 1x MES Hybridization Buffer (100 mM MES, 1M NaCl, 20mM

EDTA, 0.01% Tween-20) (all reagents from Sigma). The hybridization mix was denatured for 10 minutes at 99ºC, incubated for 5 minutes at 45ºC and spun for 5 minutes in a benchtop microcentrifuge. The microarray expression studies were performed using an Affymetrix MG-U74Av2 GeneChips (Affymetrix) for each labeled RNA sample (30 in total). The microarrays were warmed to room temperature and prehybridized in 1x hybridization buffer for 10-20 minutes at 45ºC. The prehybridization solution was removed and 150 mL of the hybridization mix was added to each array. The array and probe fragments were incubated at 45ºC overnight (16-20 hours). Following hybridization, non-specifically bound probe was removed by washing using the GeneChip Fluidics Station 400 (Affymetrix). In total, ten low stringency washes (6x SSPE, 0.01% Tween-20, 0.005% Antifoam) and four high stringency washes (100 mM MES, 0.1 M NaCl, 0.01% Tween-20, 50ºC) were performed (all reagents from Sigma). Detection of specifically bound probe was performed by incubating the arrays with SAPE (streptavidin phycoerthryin)(Molecular Probes) and biotinylated anti-streptavidin antibody (Vector Laboratories) and scanning the chips using a Gene Array Scanner (Agilent).

4. **Measurement data and specifications:**

- **Raw data:** Please see attached CEL files in Cel_and_ExpressionSummary_Data.zip.

- **Normalized and summarized data:** Please refer to the files (E1_RMA.csv, E2_RMA.csv, E3_RMA.csv) in Cel_and_ExpressionSummary_Data.zip which contains RMA-normalized data for this experiment. The columns in the spreadsheet are named based on the associated cell files (see table above). Annotations for individual probe sets are contained in MG_U74Av2_annot_csv.zip.

- **Data extraction and processing protocols:** Data was extracted using Microarray Analysis Suite 5.0 (Affymetrix). The resulting .CEL files were normalized and analyzed as described below.

- **Image scanning hardware and software:** Affymetrix Gene Array Scanner (Agilent); Microarray Analysis Suite 5.0 (Affymetrix).

- **Normalization, transformation and data selection procedures and parameters:** Data from each of the three experiments were processed on a per experiment basis. Two arrays from Experiment 3 (#15 and 29) failed quality assessment by the dChip software package[1] and were deleted from the study. The remaining chips were normalized with the robust multi-array average algorithm RMA, [2]. Differential expression was tested by independent t-tests and corrected for multiple testing using the false discovery rate procedure FDR, [3]; the FDR (*q*) was set to 0.05. Modified t-tests using the significance analysis of microarrays procedure SAM, [4] were also computed, with delta of 0.25, 0.425 and 0.54269 for experiments 1, 2 and 3, respectively. These deltas corresponded to a false positive rate of approximately 0.05. The following software packages, libraries and algorithms were used: dChip, version 1.3; R, version 1.7.1 with the Bioconductor package (Biobase, version 1.3.22, Affymetrix version 1.2.30 and RMA background

correction, version 2); S-plus, version 6.1.2 – release 1 with Array Analyzer, version 1.1.2; for FDR, the script FDR_Splus.txt can be found at http://www.math.tau.ac.il/%7eroee/index.htm; and SAM was version 1.21.

5. **Array Design:**

- Samples were run using Affymetrix MU74Av2 GeneChips. Further information regarding this commercial microarray design ca n be found at:

  http://www.affymetrix.com/support/technical/byproduct.affx?product=mgu74

- The probe sets are also described in the attached the array annotation file MG_U74Av2_annot_csv.zip.

1. Li, C. & Wong, W.H. Model-based analysis of oligonucleotide arrays: expression index computation and outlier detection. *Proc Natl Acad Sci U S A* **98**, 31-6. (2001).
2. Irizarry, R.A. et al. Summaries of Affymetrix GeneChip probe level data. *Nucleic Acids Research* **31**(2003).
3. Benjamini, Y. & Hochberg, Y. Controlling the false discovery rate: A practical and powerful approach to multiple testing. *Journal of the Royal Statistical Society Series B - Methodological* **57**, 289-300 (1995).
4. Tusher, V.G., Tibshirani, R. & Chu, G. Significance analysis of microarrays applied to the ionizing radiation response. *Proceedings of the National Academy of Science of the United States of America* **98**, 5116-5121 (2001).
